# ﻿Phylogenomics of paleoendemic lampshade spiders (Araneae, Hypochilidae, *Hypochilus*), with the description of a new species from montane California

**DOI:** 10.3897/zookeys.1086.77190

**Published:** 2022-02-17

**Authors:** Erik Ciaccio, Andrew Debray, Marshal Hedin

**Affiliations:** 1 Department of Biology, San Diego State University, San Diego, California, USA San Diego State University San Diego United States of America; 2 Department of Entomology, Plant Pathology and Nematology, University of Idaho, Idaho, USA University of Idaho Idaho United States of America; 3 Nano PharmaSolutions Inc., San Diego, California, USA Nano PharmaSolutions Inc. San Diego United States of America

**Keywords:** Conservation, mountains, multispecies coalescent, short-range endemism, Sierra Nevada, southern Appalachians, taxonomic over-splitting, ultraconserved elements

## Abstract

*Hypochilus* is a relictual lineage of Nearctic spiders distributed disjunctly across the United States in three montane regions (California, southern Rocky Mountains, southern Appalachia). Phylogenetic resolution of species relationships in *Hypochilus* has been challenging, and conserved morphology coupled with extreme genetic divergence has led to uncertain species limits in some complexes. Here, *Hypochilus* interspecies relationships have been reconstructed and cryptic speciation more critically evaluated using a combination of ultraconserved elements, mitochondrial CO1 by-catch, and morphology. Phylogenomic data strongly support the monophyly of regional clades and support a ((California, Appalachia), southern Rocky Mountains) topology. In Appalachia, five species are resolved as four lineages (*H.thorelli* Marx, 1888 and *H.coylei* Platnick, 1987 are clearly sister taxa), but the interrelationships of these four lineages remain unresolved. The Appalachian species *H.pococki* Platnick, 1987 is recovered as monophyletic but is highly genetically structured at the nuclear level. While algorithmic analyses of nuclear data indicate many species (e.g., all *H.pococki* populations as species), male morphology instead reveals striking stasis. Within the California clade, nuclear and mitochondrial lineages of *H.petrunkevitchi* Gertsch, 1958 correspond directly to drainage basins of the southern Sierra Nevada, with *H.bernardino* Catley, 1994 nested within *H.petrunkevitchi* and sister to the southernmost basin populations. Combining nuclear, mitochondrial, geographical, and morphological evidence a new species from the Tule River and Cedar Creek drainages is described, *Hypochilusxomote***sp. nov.** We also emphasize the conservation issues that face several microendemic, habitat-specialized species in this remarkable genus.

## ﻿Introduction

Discovering and delimiting cryptic species boundaries is, almost by definition, challenging. When the multispecies coalescent (**MSC**) is applied to species delimitation, species boundaries are explored by estimating gene trees and accounting for species tree/gene tree discordance using MSC models. Critical to this approach is discerning the boundary between population-level versus species-level divergence, as a core assumption of most MSC models is that species are panmictic and without population structure (Degnan and Rosenberg 2009). It is now well-established that, in systems with high natural population genetic structuring, MSC-based delimitation methods can conflate structure at the population level with divergence at the species level (Carstens et al. 2013; Sukumaran and Knowles 2017; Chambers and Hillis 2019; Mason et al. 2020). Many empirical studies indicate that, used alone, MSC methods can drastically over-split taxa (e.g., Niemiller et al. 2012; Satler et al. 2013; Hedin et al. 2015; Derkarabetian et al. 2019; Hundsdoerfer et al. 2019).

Population genetic structuring is rather ubiquitous in nature. Species with strict or semi-strict habitat or microhabitat preferences will naturally occur discontinuously over a landscape. Combine this natural habitat fragmentation with limited dispersal ability, and populations will evolve to be genetically different, to various degrees (Templeton et al. 1990; Coates et al. 2018; Marshall et al. 2021). Under some circumstances, arrays of parapatric or allopatric populations which are diverging genetically might remain morphologically quite similar, particularly when microhabitat preferences are strong (Stockman and Bond 2007; Bernardo 2011; Fišer et al. 2018). This combined suite of circumstances corresponds to what we refer to as a “no gene flow” or “non-adaptive radiation” speciation model (Gittenberger 1991; Kozak et al. 2006). Non-adaptive speciation is quite common in nature (e.g., Reilly and Wake 2015; Leavitt et al. 2007; Emata and Hedin 2016; Singhal et al. 2018; Derkarabetian et al. 2022), and challenges species delimitation. This species delimitation problem lies at one end of a spectrum of difficult scenarios for species delimitation (a high gene flow model representing an opposite, but equally challenging, scenario). Non-adaptive speciation represents a conundrum for species delimitation, as genetic data combined with many currently available models will likely over-split taxa, while other lines of evidence needed to confirm or reject this over-splitting (e.g., morphological evidence, etc.) is difficult to uncover in these same taxa (Derkarabetian et al. 2022).

**Figure 1.**
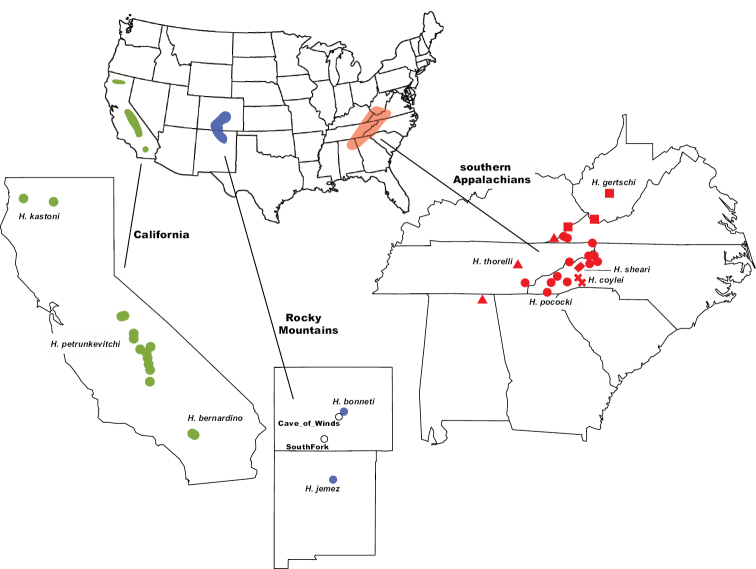
Distribution of the three geographic groups of *Hypochilus* in the mountains of California, the Rocky Mountains, and the southern Appalachians. Regional insets show the sampling locations of forty-three *Hypochilus* specimens used in genetic analyses; Appalachian species represented by different symbols.

The spider genus *Hypochilus* Marx, 1888 represents a challenging system for species delimitation, combining allopatric geographic distributions, morphological conservatism, and high genetic structuring. *Hypochilus* is a Nearctic genus representing one of two described genera in the family Hypochilidae, a family of true spiders which retain many interesting plesiomorphic traits (Forster et al. 1987; Alberti and Coyle 1991; Catley 1994). Commonly known as lampshade spiders, *Hypochilus* spiders are microhabitat specialists occurring in shaded, mesic, rock outcrop habitats (Catley 1994; Hedin and Wood 2002; Keith and Hedin 2012). *Hypochilus* includes ten described species from three disjunct montane regions: the southern Appalachians, the southern Rocky Mountains, and the California mountains (Fig. 1). Described species are mostly exclusively allopatric, and within montane regions where species are in close geographic proximity, occur in parapatric patchworks. These spiders are textbook examples of so-called short-range endemic (**SRE**) taxa, including species with naturally small geographic distributions (often defined as less than 10,000 km^2^; Harvey 2002, Harvey et al. 2011). Several *Hypochilus* species occupy severely limited geographic distributions; for example, *H.bernardino* Catley, 1994 is only known from a handful of locations in a single mountain range (San Bernardino mountains of southern California). Multiple restricted-distribution species also warrant conservation attention, particularly in the face of climate change; this conservation focus also highlights the need for rigorous species delimitation (e.g., Hedin 2015).

As spiders with extraordinarily low vagility, one would expect deeper phylogenetic relationships in *Hypochilus* to closely mirror geography, with phylogenetic predictions following geography. However, previous studies have suggested that this may be an oversimplification. Both morphological and mitochondrial data suggest that the geographically separated California and Appalachian mountain faunas are sister lineages (Catley 1994; Hedin 2001), although this inferred relationship is sensitive to varying combinations of data and analyses (Hedin 2001). Also, monophyly of the California fauna has been questioned, as both morphological and mitochondrial tree topologies sometimes recover an Appalachian clade within a larger paraphyletic Californian group (Hoffman 1963; Hedin 2001). Given the impressive geographic disjunction between these faunas (Fig. 1), such a pattern would be biogeographically compelling, if verified. Regional non-monophyly has been well-established in other north-temperate, habitat-specialized arthropods (e.g., *Brachycybe* millipedes – Brewer et al. 2012; *Sabacon* harvesters – Schönhofer et al. 2013; travunioid harvesters – Derkarabetian et al. 2018; leptonetid spiders – Ledford et al. 2021), so this biogeographic pattern is certainly possible.

As commonly found in SRE taxa, prior intraspecific genetic research in *Hypochilus* has revealed ubiquitous and extensive genetic structuring. In Appalachia, extreme mitochondrial genetic divergence occurs within and among five described species over small geographic distances (Hedin 2001; Hedin and Wood 2002; Keith and Hedin 2012). Within *H.pococki* Platnick, 1987, recovered as paraphyletic on mitochondrial gene trees, highly divergent, geographically cohesive “microclades” have been discovered (Keith and Hedin 2012). In California, mitochondrial CO1 sequences for *H.petrunkevitchi* Gertsch, 1958 from the Merced versus Kaweah River basins reveal extreme intraspecific genetic divergences (> 15% divergent; Hedin 2001). Past mitochondrial studies have attributed extensive genetic structuring to limited female-biased gene flow (Hedin and Wood 2002; Keith and Hedin 2012), but whether such genetic patterns extend to the nuclear genome and are less pronounced because of male-based gene flow remains unknown.

In this research we used phylogenomic data to resolve *Hypochilus* species relationships within and among montane regions. We also explored putative cryptic diversification within Appalachian *H.pococki* and Californian *H.petrunkevitchi*. In Appalachia, previous mitochondrial-based species delimitation using a Generalized Mixed Yule Coalescent (GMYC) model (Fujisawa and Barraclough 2013) appears to severely over-split species (Keith and Hedin 2012), likely due to strong genetic structuring. We asked whether similar results applied to nuclear single nucleotide polymorphism (**SNP**) datasets derived from ultraconserved elements (**UCEs**), using additional MSC methods (Bayes Factor Delimitation and TR2 rooted triplet analysis). We combine these molecular data and analyses with SEM images of the male pedipalp for large samples of *H.pococki* and *H.petrunkevitchi*, and gather UCE CO1 mitochondrial “by-catch” data for *H.petrunkevitchi*. Based on evidence derived from a combination of nuclear phylogenomic and mitochondrial data, geography, and adult male and female morphology, we describe a new SRE species from the southern Sierra Nevada of California.

## ﻿Materials and methods

### ﻿Molecular sampling

Specimens representing the genus *Ectatosticta* Simon, 1892 from China, the sister genus to *Hypochilus* (and the only other hypochilid genus described), were used to root all phylogenies (UCE data from Ramírez et al. 2021). The monophyly of the family, and two constituent genera, is strongly supported by both morphology (Forster et al. 1987; Alberti and Coyle 1991; Catley 1994; Li et al. 2021) and phylogenomics (Fernández et al. 2018; Ramírez et al. 2021). Phylogenomic data were gathered for forty-three *Hypochilus* specimens, representing all ten described species (see Suppl. material 1; Fig. 1). A priori species identifications were based on male morphology in combination with geography (Forster et al. 1987; Catley 1994), and we included many samples from prior genetic research (Keith and Hedin 2012). Multiple specimens per species were sampled, chosen to maximize the breadth of geographic coverage within species (Fig. 1). Our southern Rocky Mountain samples included collections from near the respective type localities (Gertsch 1964; Catley 1994) of both *H.bonneti* Gertsch, 1964 and *H.jemez* Catley, 1994, plus two geographically intermediate populations from southern Colorado of uncertain species identity. For species delimitation focal taxa (*H.pococki* and *H.petrunkevitchi*), larger sample sizes were used to obtain more fine-scale genetic data on potentially cryptic species (Fig. 1). For *H.pococki*, sampling included representatives of the five mitochondrial haplogroups identified in Keith and Hedin (2012). For *H.petrunkevitchi*, specimens were sampled from multiple drainage basins, including the Merced River (**YOSE**), San Joaquin River (**SAN**), Kings River (**KING**), Kaweah River (**KAW**), Tule River (**TULE**), and Cedar Creek (**CEDAR**) drainages.

### ﻿UCE data collection

For almost all specimens (with tissues stored at -80 °C), DNA extraction was performed using a Qiagen DNEasy kit from leg tissues. Sequence capture libraries were prepared using an ultraconserved elements capture protocol for arachnids (Starrett et al. 2017; Hedin et al. 2019), with arachnid probes designed by Faircloth (2017). Sequencing was done at the Brigham Young University DNA Sequencing Center on an Illumina HiSeq 2500 150 cycle paired-end sequencing platform. Published data for two *H.pococki* specimens (H595 and H232, from Starrett et al. 2017), one *H.kastoni* Platnick, 1987 (G2519, Hedin et al. 2019), and *Ectatosticta* (Ramírez et al. 2021) were used from prior studies. Raw reads were filtered using the illumiprocessor wrapper (Faircloth 2013) within PHYLUCE v1.6 (Faircloth 2016), after which cleaned reads were assembled using Trinity v2.0.6 (Grabherr et al. 2011) and Velvet v1.0.19 (Zerbino and Birney 2008) on the HPC Cluster at UC Riverside. These assemblies were combined and resulting contigs were matched to probes with minimum identity and minimum coverage values of 80 (--min-identity 80 --min-coverage 80). UCE loci were aligned and trimmed within PHYLUCE using MAFFT (Katoh and Standley 2013) and Gblocks (Castresana 2000; Talavera and Castresana 2007) with relatively liberal settings for GBLOCKS (--b1 0.5 --b2 0.5 --b3 10 --b4 8).

A 50 percent occupancy matrix (623 loci) was generated from the pipeline above (here called the “unfiltered” matrix). A second matrix was further filtered to remove duplicate and potentially non-homologous sequences. Previous work has shown that arthropod UCEs are mostly located within exonic regions (Bossert and Danforth 2018), and the arachnid probe set is no exception (Hedin et al. 2019). In fact, the arachnid probe set can target separate exons from the same protein as separate loci (Hedin et al. 2019), perhaps violating assumptions such as independence and linkage of loci. An annotated list of the arachnid UCEs from Hedin et al. (2019) was used to identify duplicate loci. A total of 73 duplicate loci was found, with the longest locus in each instance retained while the rest were discarded. Using the annotated list, those loci identified by Hedin et al. (2019) as including potential paralogs were also identified and discarded (*n* = 2), leaving a matrix containing a total of 550 loci. Finally, this filtered dataset was trimmed again with more stringent Gblocks settings (--b1 0.5 --b2 0.85 --b3 4 --b4 8), resulting in a “filtered and trimmed” matrix. This last step was conducted to isolate as much of the purely exonic region as possible for each locus.

### ﻿Generic-level phylogenomics

Interspecific relationships were reconstructed using all three UCE data matrices (“unfiltered”, “filtered”, and “filtered and trimmed”), utilizing both concatenation and coalescent-model approaches. Data were partitioned by locus, with optimal models selected using PartitionFinder2 (Lanfear et al. 2012) on the CIPRES portal HPC Cluster. Concatenated maximum likelihood trees were reconstructed using RAxML v8.2.12 (Stamatakis 2014) and IQ-TREE v1.5.0 (Nguyen et al. 2015). Using IQ-TREE 2 we also calculated gene (**gCF**) and site concordance (**sCF**) factors. For every node of a reference tree, gCF can be defined as the percentage of “decisive” gene trees containing that node, while sCF can be defined as the percentage of decisive sites (in an alignment) supporting a node (Minh et al. 2018). The latter support metric is particularly useful when individual gene trees are uncertain, perhaps because individual alignments are short. Concordance factor calculations were performed using the topology from IQ-TREE which has an identical topology to the RAxML tree and mostly agrees with the SVDquartets reconstruction (see Results). The latter is a coalescent-model topology reconstructed using SVDquartets v1.0 (Chifman and Kubatko 2014) implemented in PAUP* (Swofford 2003), set for 1M quartets with 500 bootstrap replicates for all runs.

### ﻿Nuclear species delimitation

Nuclear single nucleotide polymorphism (**SNP**) data were extracted from UCE loci following the methods of Harvey et al. (2016) and a combination of tools and methods from vcf tools (Danecek et al. 2011) and a modified version of the best practices approach for variant isolation with GATK v4.0.0.0 (Van der Auwera et al. 2013). Separate datasets were created for Californian *H.petrunkevitchi* plus *H.bernardino* specimens, and for samples of *H.pococki*. These matrices started with cleaned read data containing only relevant samples and used a highest coverage reference specimen (H_petrunkevitchi_G2543, H_pococki_H551). Genetic structure and sample clustering were explored using k-means clustering in STRUCTURE v2.3.4 (Pritchard et al. 2000), with unlinked SNPs. Multiple K values (K = 1–10) were run for 100,000 generations with each K value replicated 10 times. Optimal K values were determined following Pritchard et al. (2000) and Evanno et al. (2005), using the online resource CLUMPAK (Kopelman et al. 2015).

Using multiple data sources (phylogenomic results, STRUCTURE results, geography for *H.petrunkevitchi*, and mitochondrial haplogroup membership for *H.pococki*), alternative species models were generated and compared using nuclear SNP datasets in the program SNAPP (Tables 1, 2; Bryant et al. 2012). These analyses also included outgroup data (other *Hypochilus* species), allowing us to test hypotheses of *H.pococki* as a single species (current taxonomy) and *H.petrunkevitchi* lumped with *H.bernardino* (see Tables 1, 2). The averaged marginal likelihoods of duplicate runs were compared for alternative species models using Bayes Factor Delimitation for genomic data (*BFD) (Leaché et al. 2014); we followed the recommendations of Kass and Raftery (1995) in interpreting Bayes factor values.

**Table 1.** Alternative species model comparison results for *H.petrunkevitchi*, from SNAPP. Alternative models were compared to current taxonomy and ranked with 1 as the most favorable and 5 as the least. Bayes factors were calculated as (BF = 2 × (model 1 – model 2)) where negative values represent support for model 2 (alternative model) and positive values are support for the null model (current taxonomy).

Model	Species	Partitioning	MLE	MLE 2	BF	Rank
Every Tip	16	Every specimen as a species	-105	-105.2	-7056	1
Basins	8	*H.kastoni*, *H.bernardino*, CEDAR, TULE, KAW, KING, SAN, YOSE	-1781	-1781	-3704	2
STRUCTURE	6	*H.kastoni*, *H.bernardino*, TULE+CEDAR, KAW+KING, SAN, YOSE	-1939	-1939	-3388	3
Current Taxonomy	3	*H.kastoni*, *H.petrunkevitchi*, *H.bernardino*	-3633	-3633	-	4
Collapse	2	*H.kastoni*, *H.petrunkevitchi + H.bernardino*	-4223	-4223	1180	5

A rooted triplet species delimitation approach was also implemented using the Python2 compatible version of the program TR2 (Fujisawa et al. 2016). Here, nuclear gene trees are decomposed into partially rooted triplets and congruence is assessed among triplet topologies using a likelihood model testing framework. Input gene trees were constructed in RAxML using rapid bootstrap analysis (-f a) with 200 bootstrap replicates for each gene tree with 550 UCE loci from the “filtered and trimmed” dataset. With the intent to detect patterns of increasing support for increasing species number (i.e., over-splitting), models in which every tip was categorized as a species were included in both SNAPP and TR2 runs.

**Table 2.** SNAPP results for *H.pococki*. Models were compared to current taxonomy and ranked with 1 as the most favorable and 4 as the least.

Model	Species	Partitioning	MLE	MLE 2	BF	Rank
Every Tip	16	Every specimen as a species	-62.9	-62.8	-5030	1
Mitochondrial	6	*H.thorelli*, WEST+bone+alark, CENT, VA, ELK, NE	-3917	-3917	-4462	2
STRUCTURE	5	*H.thorelli*, WEST+bone+alark, CENT, ELK+NE, VA	-4338	-4340	-3620	3
Current Taxonomy	2	*H.thorelli*, *H.pococki*	-6148.9	-6148	-	4

### ﻿CO1 phylogeny, distances, and GMYC

Mitochondrial Cytochrome c oxidase subunit I (CO1) data for the California taxa were captured from UCE “by-catch” for purposes of phylogenetic and distance analyses, particularly considering the extreme CO1 distances observed in Hedin (2001). A consensus reference sequence was created from specimens of *H.petrunkevitchi* and *H.bernardino* (from Hedin 2001), which was then used as a custom database for a BLASTN search for extracting CO1 sequences from UCE contigs. The BLASTN search and subsequent alignment was performed using Geneious Prime (2020.2). Sequence alignments were partitioned by codon position using MODELFINDER (Kalyaanamoorthy et al. 2017) and a phylogeny was constructed using IQ-TREE with 1000 ultrafast bootstrap replicates. A pairwise mitochondrial distance matrix was generated in PAUP* (Swofford 2003) using a Kimura two-parameter model of nucleotide substitution (Kimura 1980).

A Generalized Mixed Yule Coalescent (**GMYC**) model was used to algorithmically delimit Californian species using CO1 data, in a manner similar to the approach of Keith and Hedin (2012) for Appalachian *H.pococki*. A GMYC model assumes a difference in intra and inter-specific branching patterns under maximum likelihood and estimates a threshold for the transition from intraspecific (a coalescent process) to interspecific (speciation) branching on an ultrametric tree. Both a single threshold and multiple thresholds can be estimated in different approaches with the model. To this end, an ultrametric CO1 tree was generated using the *chronopl* function from the APE library in R version 4.0. 2 (Paradis and Schliep 2019), with both single and multiple threshold models performed on the GMYC web server (https://species.h-its.org/gmyc/; Fujisawa and Barraclough 2013).

### ﻿Morphological study

Although *Hypochilus* is a strongly morphologically conserved genus, current species were described and are diagnosed using subtle morphological variation, mostly in male genitalia (Hoffman 1963; Forster et al. 1987; Catley 1994). Using SEM we examined male palpal morphology for California taxa (*H.kastoni*, *H.bernardino*, multiple lineages of *H.petrunkevitchi*), and for all primary lineages of *H.pococki*. We lacked adult males for the southern Rocky Mountain populations of uncertain species identity, so could not study them at this time. Male pedipalps preserved in 80% EtOH were transferred to pure EtOH (200 PF, ≥ 99.5%) for at least 10 min. Following EtOH dehydration, samples were placed in a multilayered sample holder and dried to the critical point using a Tousimis SAMDRI790. Transitional and intermediate fluids used were CO_2_(l) and pure EtOH, respectively. Dried palps were mounted on aluminum stubs fitted with sticky carbon conductive spectro-grade tabs. Following mounting, samples were run through an EMS Quorum Q150T sputter coater and covered with 6 nm platinum nanoparticles. SEM micrographs of retrolateral and prolateral views were taken using a vertical stage on an FEI Quanta 450 FEG. Female spermathecal organs were imaged using a Visionary Digital Imaging System, comprising a Canon EOS 5D Mk II DSLR mounted to an Infinity Optics microscope tube. Spermathecal organs were dissected from specimens using fine forceps, immersed for 2–5 minutes in BioQuip specimen clearing fluid (http://www.bioquip.com), then imaged in this fluid on depression slides.

For taxonomic descriptions, morphological measurement details follow Catley (1994: figs 1–4):

**PTW/PTL** maximum width of male pedipalpal tibia in retrolateral view/length of tibia in retrolateral view;

**CdL** male palpal conductor length in retrolateral view;

**AME** diameter of anterior median eye pupil;

**PTaL** length of male palpal tarsus in retrolateral view;

**CTpr** number of promarginal cheliceral teeth;

**CTre** number of retromarginal cheliceral teeth.

Measurements were taken from alcohol-preserved specimens using an Olympus SZ40 dissecting microscope fitted with an ocular micrometer, and converted to millimeters; raw measurements are provided in Suppl. material 2, and summarized in Table 5.

## ﻿Results

### ﻿UCE processing and generic-level phylogenomics

Original UCE raw reads have been submitted to the SRA (PRJNA760946), with summary statistics presented in the Suppl. material 1. Data matrices and resulting .tre files are deposited at Dryad (https://doi.org/10.5061/dryad.g1jwstqsd). The “unfiltered” matrix included 623 UCE loci with an average length of 783 base pairs (**bp**) and a concatenated length of 734,881 bp (189,387 parsimony informative (**PI**) sites). The “filtered” matrix included 550 loci with an average length 921 bp and concatenated length of 506,689 bp (145,037 PI sites), while the “filtered and trimmed” matrix contained 550 loci with an average length of 591 bp and concatenated length of 325,452 bp (81,911 PI).

**Figure 2.**
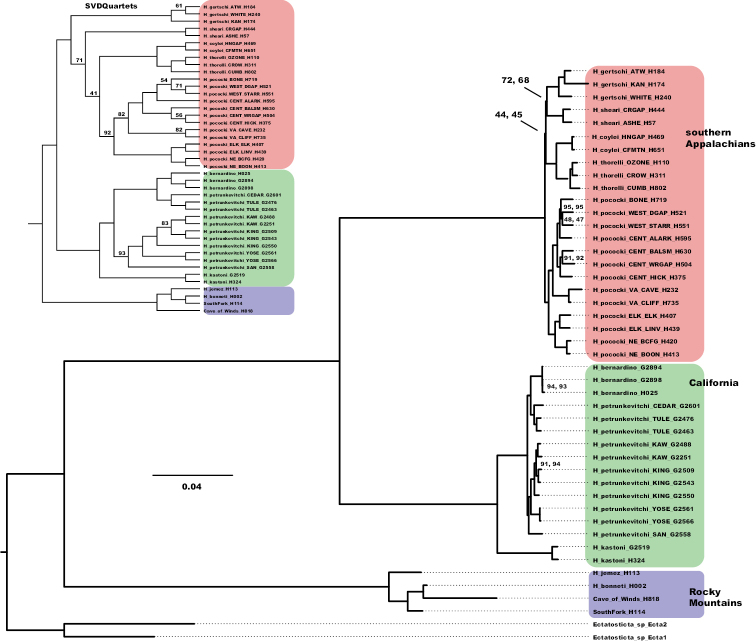
UCE phylogeny. Phylogeny reconstructed from partitioned RAxML analysis of “filtered and trimmed” UCE matrix. Bootstrap support values are 100 for all nodes unless otherwise indicated; second support values from IQ-TREE. Inset – SVDquartets UCE tree with bootstrap support values less than 95 shown.

**Figure 3.**
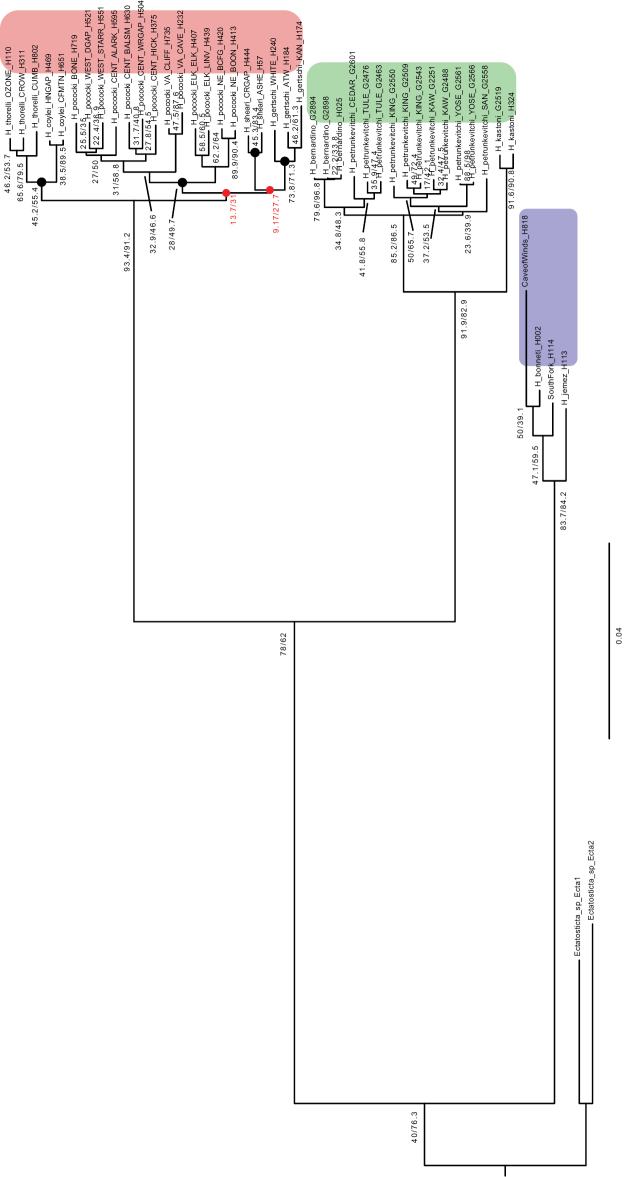
Concordance Factor values. Phylogeny reconstructed from partitioned RAxML analysis of “filtered and trimmed” UCE matrix, with gCF / sCF values. The lowest gCF and sCF values, for two unresolved nodes which collapse to a four lineage polytomy within the Appalachian clade, are highlighted by red text.

Concatenated ML and SVDquartets analyses of the above three matrices recover nearly identical *Hypochilus* relationships, except for some nodes in the Appalachian and Rocky Mountain clades (Figs 2, 3; https://doi.org/10.5061/dryad.g1jwstqsd). These analyses confirm regional faunas as monophyletic, rejecting CA paraphyly, and recover Appalachia and California clades as sister taxa. Within Appalachia, a sister species relationship between the geographically disjunct *H.thorelli* Marx, 1888 and *H.coylei* Platnick, 1987 is strongly supported across all analyses, consistent with mitochondrial evidence (Hedin 2001; Keith and Hedin 2012), but contrary to morphological evidence which groups *H.coylei* with the geographically adjacent *H.sheari* Platnick, 1987 (Huff and Coyle 1992; Catley 1994). The monophyly of all currently recognized Appalachian species is supported, contradicting mitochondrial results which had previously suggested *H.pococki* paraphyly. Certain parts of the Appalachian topology include low bootstrap values, low gene and site concordance values, and discordant topologies among analyses. Whereas ML analyses place *H.pococki* as sister to other Appalachian species, SVDquartets nests *H.pococki* well within the clade with *H.gertschi* Hoffman, 1963 as sister to other Appalachian species (Fig. 2). A California clade is consistently recovered, with *H.kastoni* sister to remaining lineages. *Hypochilusbernardino* is nested prominently within *H.petrunkevitchi*, and for this reason analyses examining species boundaries included samples of all three species (see below).

### ﻿Nuclear species delimitation

**Figure 4.**
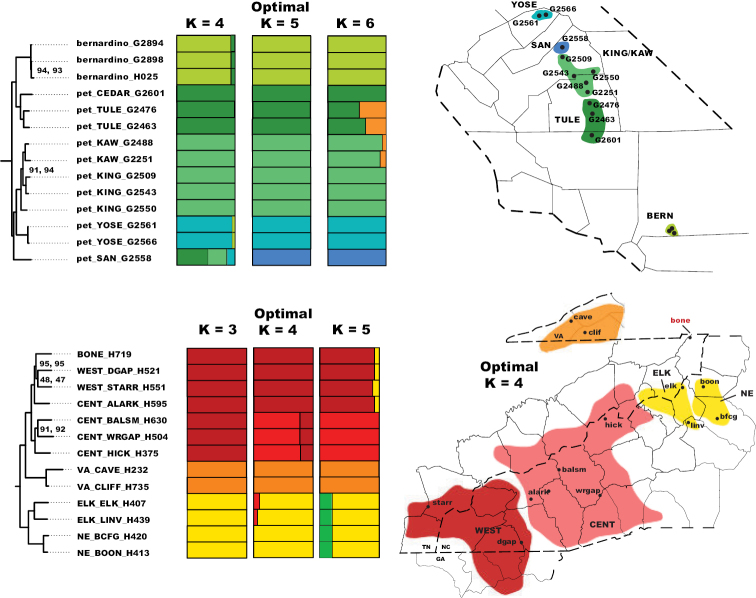
UCE STRUCTURE results, with optimal and suboptimal K clusters shown in relation to UCE RAxML phylogeny and geography. For *H.pococki*, the distribution of mitochondrial “microclades” follows Keith and Hedin (2012).

Nuclear SNP datasets included 670 unlinked SNPs for the California sample (allowing 20% missing data) and 655 unlinked SNPs for the Appalachian sample (19% missing data). Overall, STRUCTURE analyses reveal strong population structure for both samples, with inferred genetic clusters congruent with phylogenomic clades (Fig. 4). Within California there is little evidence for mixed ancestry, with genetic populations of *H.petrunkevitchi* also appearing to be structured by river basin (Fig. 4). Best K as determined by the Pritchard et al. (2000) method is decisive for K = 5 while the Evanno method is less conclusive, supporting a scenario of K = 2 with almost equal but slightly lower support for K = 4 (https://doi.org/10.5061/dryad.g1jwstqsd). When *H.kastoni* samples were included in STRUCTURE runs, the Pritchard method recovered the same population structure scheme with the addition of a separate *H.kastoni* “population”, while the Evanno method recovered stronger support for K = 2 in which *H.kastoni* and *H.bernardino* comprised a single genetic population. Because we viewed this latter K = 2 result as spurious (see Janes et al. 2017), we generally preferred optimal K values as inferred by the Pritchard method.

There is more evidence for mixed ancestry in *H.pococki* STRUCTURE analyses, using a best K from both Pritchard (K = 4) and Evanno (K = 3) methods. The Elk and Northeast “microclades” (ELK and NE) are lumped as a single genetic population, while one member from the mitochondrial Central (CENT) group clusters with the WEST population (Fig. 4), discordant with previously delineated mitochondrial groups (Keith and Hedin 2012). The geographically isolated BONE population, not sampled in previous mitochondrial studies, clusters with disjunct WEST specimens (Fig. 4).

Alternative species hypothesis models were generated and compared using SNAPP and TR2 for both *H.petrunkevitchi* (five models) and *H.pococki* (four models). Pritchard-based best K schemes were used for the STRUCTURE derived species models. The most-favored SNAPP model for *H.petrunkevitchi* (Table 1) is one where every tip represents a species; the next best supported model is the “Basins”, 8-taxon model. SNAPP results for *H.pococki* were similar and favored every tip as a species as the best model, with a pattern of more speciose models being more favored (Table 2). The rooted triplet TR2 approach also found this pattern of favoring more species-rich models over current taxonomy with the most species-rich model, every tip as a distinct taxon, being the most favored (Tables 3, 4).

**Table 3.** TR2 results for *H.petrunkevitchi*; ranking of the models with 1 being the most favored and 6 being the least favored.

Model	Species	Partitioning	Score	Rank
Every Tip	16	Every specimen as a species	171.62	1
Basins	8	*H.kastoni*, *H.bernardino*, TULE, CEDAR, KAW, KING, SAN, YOSE	213.95	2
STRUCTURE	6	*H.kastoni*, *H.bernardino*, TULE+CEDAR, KAW+KING, YOSE, SAN	348.23	3
Current Taxonomy	3	*H.kastoni*, *H.petrunkevitchi*, *H.bernardino*	9334.94	4
Collapse	2	*H.kastoni*, *H.petrunkevitchi + H.bernardino*	25926.79	5
One species	1	*H.kastoni + H.petrunkevitchi + H.bernardino*	30938.48	6

### ﻿CO1 phylogeny, distances, and GMYC

**Figure 5.**
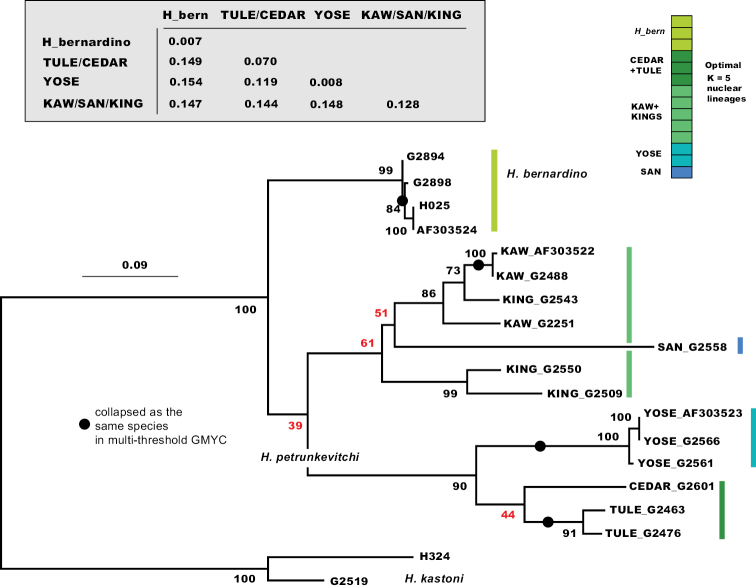
Maximum likelihood mitochondrial tree. Black circles designate clusters of sequences collapsed as the *same* species in multi-threshold GMYC analyses; all other branches supported as separate species (e.g., n = 13 for multi-threshold model). **Inset** – K2P distances within and among primary mitochondrial lineages.

The CO1 by-catch phylogeny, using *H.kastoni* and *H.bernardino* as possible outgroups, shows strong support (BP = 100) for a clade including *H.bernardino* and *H.petrunkevitchi* together (Fig. 5). Within this clade, recovered mitochondrial lineages are consistent with UCE optimal K = 5 STRUCTURE lineages, but the interrelationships of these mitochondrial lineages are not resolved. Considering this phylogenetic uncertainty (i.e., collapsing poorly-resolved nodes), the mitochondrial results are not strictly inconsistent with nuclear results. Pairwise mitochondrial distance values are extremely high among primary lineages (Fig. 5 inset), ranging from 12%–15%. Divergence values within lineages are lower, except for the combined SAN + KING + KAW lineage (> 12% divergence); this obviously reflects significant mitochondrial divergence across drainage basins within this more broadly-distributed lineage. Similarly, there is evidence for structuring across drainage basins within the combined TULE + CEDAR lineage (Fig. 5 inset). As shown previously for Appalachian *H.pococki* (Keith and Hedin 2012), implementation of a GMYC model using CO1 data appears to severely over-split species. Specimens from the same geographic location are collapsed as the same species, but all unique geographic locations are delimited as distinct species (multi = 13, single = 14; Fig. 5 inset).

**Table 4.** TR2 results for *H.pococki*; ranking of the models with 1 being the most favored and 5 being the least favored.

Model	Species	Partitioning	score	Rank
Every Tip	16	Every specimen as a species	344.10	1
Mitochondrial	7 (*includes BONE as separate lineage)	*H.thorelli*, WEST, CENT, ELK, NE, VA, BONE	366.66	2
STRUCTURE	5	*H.thorelli*, WEST+Bone+Alark, CENT, ELK+NE, VA	819.85	3
Current	2	*H.thorelli*, *H.pococki*	9930.73	4
Collapse	1	*H.thorelli + H.pococki*	17961.09	5

### ﻿Morphology

Morphological data for *H.pococki* and California taxa are presented below in the Discussion and Taxonomy sections, respectively.

## ﻿Discussion

### ﻿Broad-scale phylogenomics and biogeography

Our results confirm the Catley (1994) hypothesis of a sister relationship between highly disjunct California and Appalachian faunas, sister to a more geographically central Rocky Mountain clade. Support was unequivocal for this topology, recovered in all analyses from all UCE matrices (Figs 2, 3; https://doi.org/10.5061/dryad.g1jwstqsd). These regional relationships in *Hypochilus* are contrary to patterns seen in the salamander genus *Aneides*, a taxon which also includes montane-associated species from California, the southern Rocky Mountains, and Appalachia. Molecular phylogenetic research in *Aneides* has recovered a sister relationship between California and southern Rocky Mountain species, sister to Appalachian taxa (Vieites et al. 2007). Inter-regional divergences in *Aneides* are estimated to have occurred roughly in the timeframe spanning the Eocene to the Oligocene, perhaps coincident with periods of global warming allowing for intercontinental dispersal events (Vieites et al. 2007).

We hypothesize that *Hypochilus* has a more ancient history, and that this timing difference might also explain the unique phylogenetic topology seen in *Hypochilus*. Divergence time estimates for *Hypochilus* are hindered by a lack of direct fossil evidence, with current age estimates for the genus derived from broader examinations of diversification dates for spiders. For example, using published transcriptome data, Magalhaes et al. (2020) estimated divergence between *H.gertschi* and *H.pococki* (both Appalachian taxa) during the early Paleogene, with a very large confidence interval. Given approximately polytomous relationships within Appalachia (see below), this point estimate would correspond to a crown group age for the Appalachian radiation, and thus implies older divergences at the base of *Hypochilus*, perhaps during the Cretaceous. We note here that many other non-entelegyne araneomorph spider lineages are at least this ancient (both within extant families and sometimes within extant genera), as estimated from molecular clock analyses (e.g., sicariids – Magalhaes et al. 2019; leptonetids – Ledford et al. 2021), but also known directly from Upper Cretaceous Burmese amber fossils (e.g., psilodercids – Magalhaes et al. 2021). Also, the combination of Cretaceous fossil evidence in the context of living spider families suggests that non-entelygyne araneomorph lineages (akin to *Hypochilus* and Hypochilidae) dominated spider diversity at this time (Wunderlich 2008; Magalhaes et al. 2020).

Hypothesized Cretaceous-age divergences for *Hypochilus* are complicated by the presence of the Western Interior Seaway of North America, a major transcontinental marine barrier in place from ~ 105–65 mya (Blakey and Ranney 2017). Although an east / west vicariance hypothesis associated with the Western Interior Seaway seems attractive, the Rocky Mountain orogeny took place after the withdrawal of the Western Interior Seaway (Blakey and Ranney 2017). We speculate that *Hypochilus* is either much older than imagined (ages exceeding 105 mya), or that diversification (and dispersal) was spurred soon after the withdrawal of the Western Interior Seaway. Lacking the discovery of relevant fossils, future work could aim to more precisely estimate rates of nuclear gene molecular evolution in *Hypochilus*, in order to better understand the origin and timing of *Hypochilus* diversification events. Also, inclusion of a transcriptome representing the Rocky Mountain clade could be incorporated into the well-calibrated Magalhaes et al. (2020) dataset, allowing for a crown group age estimate for the entire genus.

### ﻿Appalachian diversification and potential cryptic species

Within Appalachia, nuclear UCE data strongly support a monophyletic *H.pococki*, contra mitochondrial paraphyly as in Keith and Hedin (2012). Although all currently described species in Appalachia are recovered as monophyletic, and the geographically disjunct *H.thorelli* and *H.coylei* are strongly supported as sister species (see also Hedin 2001; Keith and Hedin 2012), our nuclear datasets otherwise do not resolve species relationships, with an overall topology consistent with a four-lineage polytomy. Gene and site concordance factor values at two key unresolved interspecific nodes take lower values than seen anywhere else in *Hypochilus*, including all nodes within species (Fig. 3). Gene CF values are 9–13 for these nodes, meaning that only ~ 10% of the UCE alignments support these nodes. Site CF values that hover around minimum values (30%) illustrate that the data are essentially equivocal regarding three possible resolutions of a quartet for both of these unresolved nodes (Lanfear 2018). This incongruence and lack of resolution possibly points to a non-adaptive radiation where lineages became rapidly isolated from one another due to environmental factors, but the nature of incongruence requires more study.

Nuclear STRUCTURE results confirm distinct genetic groups within *H.pococki* (Fig. 4); however, these genetic groups do not correspond exactly to the previously described mitochondrial “microclades”. In particular, the Alarka Mountain specimen from the mitochondrial Central (CENT) clade of Keith and Hedin (2012) instead groups with the nuclear WEST genetic cluster (Fig. 4). This is important because the geographic boundaries of previously defined mitochondrial clades were hypothesized to coincide with riverine barriers (e.g., the Little Tennessee River separating the CENT versus WEST mitochondrial clades, etc.; see Keith and Hedin 2012: fig. 2). In fact, most phylogeographic studies in the southern Appalachians have primarily relied upon mitochondrial evidence to define geographic groupings (e.g., references in Keith and Hedin 2012). Future studies that include dense geographic sampling of nuclear lineages will be important here, with *H.pococki* representing a prime candidate. More generally, if gene flow across cryptic lineages is promoting mitonuclear discordance, this system might provide interesting insight into how cryptic lineages interact at areas of contact. Also, areas of parapatric contact can be used to understand the degree to which gene flow is restricted across cryptic lineage boundaries, providing strong and direct tests of species status (see Singhal et al. 2018).

**Figure 6.**
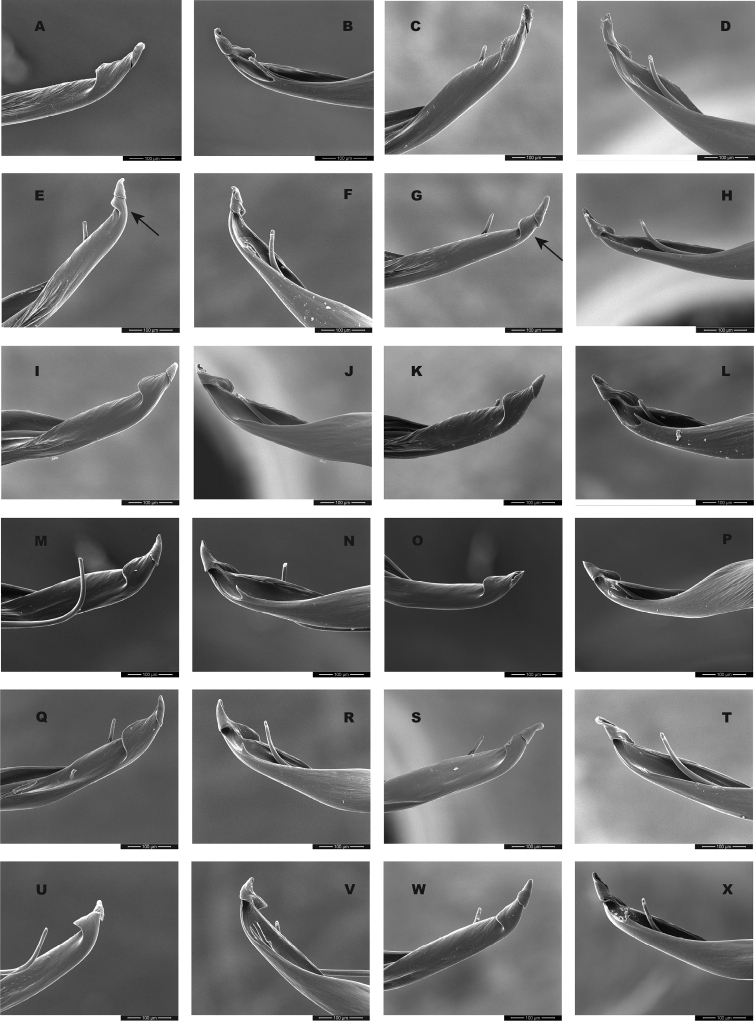
*H.pococki* male palp (conductor) comparison. For each specimen, left panel = prolateral view, right panel = retrolateral view. *NE lineage***A, B** Green Mtn (MCH 01_162) **C, D** Boone Fork (MCH 01_159); *ELK lineage***E, F** Elk River (MCH 01_155) **G, H** Linville Gorge (MCH 01_165). Shorter secondary coil for ELK specimens highlighted by arrows; *VA lineage***I, J** Cliff Mtn (MCH 04_028) **K, L** Guest River (MCH 04_027); *CENT lineage***M, N** Hickory (MCH 01_144) **O, P** Wagon Road Gap (MCH 01_181); *WEST lineage***Q, R** Alarka (MCH 02_168) **S, T** Starr Mtn (MCH 02_156) **U, V** Backbone Rock (MCH 04_025) **W, X** Chunky Gal Mtn (MCH 02_142). Detailed specimen information provided in Suppl. material 2.

**Figure 7.**
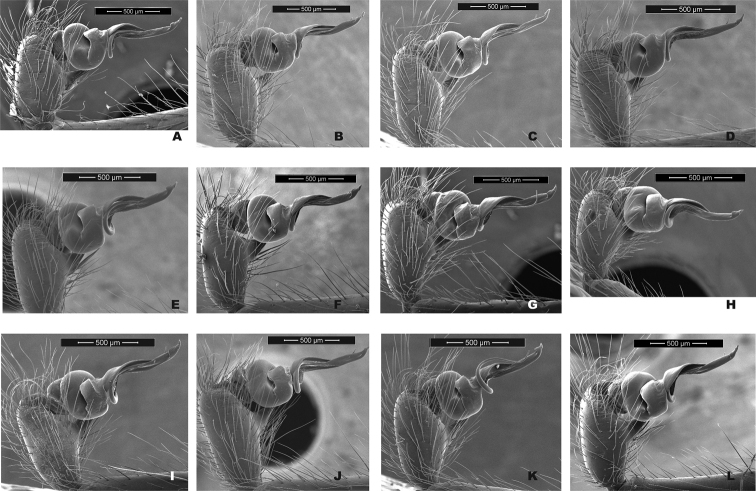
*H.pococki* male palp comparison, prolateral views. *NE lineage***A** Green Mtn (MCH 01_162) **B** Boone Fork (MCH 01_159); *ELK lineage***C** Elk River (MCH 01_155) **D** Linville Gorge (MCH 01_165); *VA lineage***E** Cliff Mtn (MCH 04_028) **F** Guest River (MCH 04_027); *CENT lineage***G** Hickory (MCH 01_144) **H** Wagon Road Gap (MCH 01_181); *WEST lineage***I** Alarka (MCH 02_168) **J** Starr Mtn (MCH 02_156) **K** Backbone Rock (MCH 04_025) **L** Chunky Gal Mtn (MCH 02_142). Detailed specimen information provided in Suppl. material 2.

**Figure 8.**
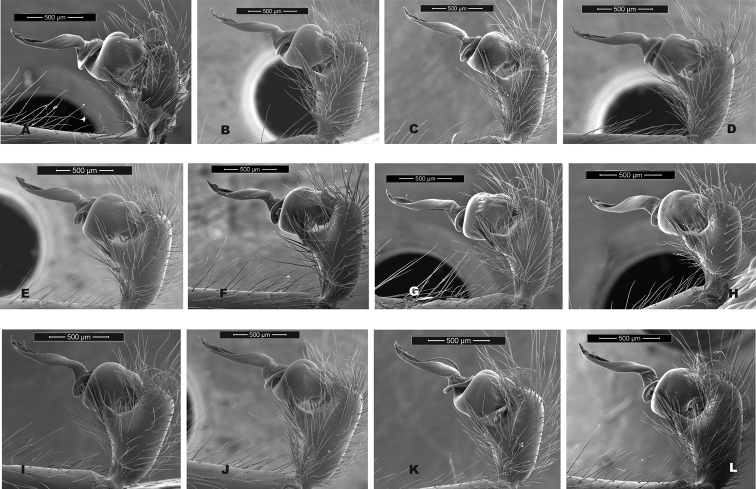
*H.pococki* male palp comparison, retrolateral views. *NE lineage***A** Green Mtn (MCH 01_162) **B** Boone Fork (MCH 01_159); *ELK lineage***C** Elk River (MCH 01_155) **D** Linville Gorge (MCH 01_165); *VA lineage***E** Cliff Mtn (MCH 04_028) **F** Guest River (MCH 04_027); *CENT lineage***G** Hickory (MCH 01_144) **H** Wagon Road Gap (MCH 01_181); *WEST lineage***I** Alarka (MCH 02_168) **J** Starr Mtn (MCH 02_156) **K** Backbone Rock (MCH 04_025) **L** Chunky Gal Mtn (MCH 02_142). Detailed specimen information provided in Suppl. material 2.

Although SNAPP and TR2 show higher support for increasing species numbers within *H.pococki* (i.e., a many species hypothesis), nuclear STRUCTURE results and consideration of male pedipalp morphology suggest more conservative species numbers. In their diagnosis, Forster et al. (1987) stated that *H.pococki* males “*can be recognized by the flaplike tip of the palpal conductor*”. We thus focused particular attention on this structure when searching for morphological differences that might distinguish primary genomic lineages (e.g., VA, ELK + NE, CENT, WEST), and included representatives of all such lineages in our SEM surveys. We did not examine female variation (e.g., in spermathecal morphology), but this is another character system to search for morphological differentiation. We observed minimal differences in male palpal morphology across *H.pococki* populations and genomic lineages (Figs 6–8). One possible difference is the shorter secondary coil of the conductor tip observed in ELK specimens (Fig. 6), but we note that ELK itself is well nested within the primary K = 4 lineages (Fig. 4). The discord between nuclear genomic data (and analytical results) which suggest many species, versus morphology which suggests few to one species, is a conspicuous example of the cryptic species challenge, and also focuses attention on patterns of morphological stasis. Despite high genomic divergences and ample evolutionary time, morphological change in *Hypochilus* remains conservative. We might expect conserved *Hypochilus* somatic morphology because of selective constraints on both niche evolution and morphological differentiation, under a model of phylogenetic niche conservatism (Keith and Hedin 2012; Fišer et al. 2018). The fact that we also observe similar conservatism in genitalia, where at least genetic drift in isolated populations is expected, is compelling.

### ﻿A new *Hypochilus* species from montane California

**Figure 9.**
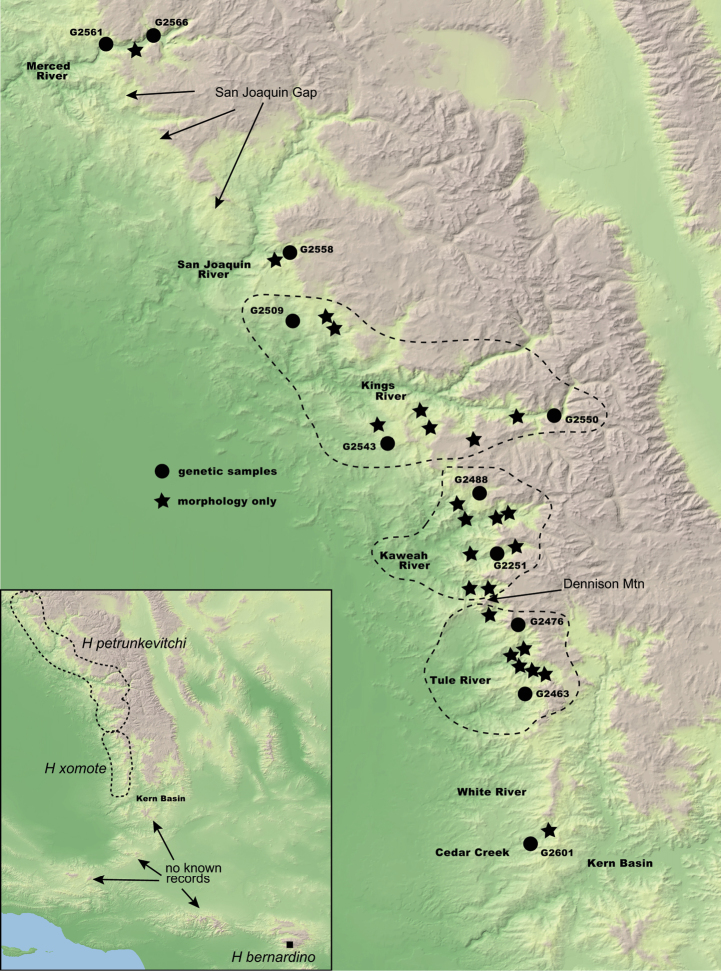
Southern Sierra Nevada topography map with genetic and morphological sample locations (see Suppl. material 1 and Suppl. material 2). Geographic gaps and other notable geographic features mentioned in the text are highlighted.

Both nuclear and mitochondrial genetic structuring is very prominent in the California region, and our analyses show that this structure generally follows a pattern of relatedness by drainage basin (Figs 4, 5, 9). The observed divergence within *H.petrunkevitchi* was not surprising as it has previously been noted as having high levels of intraspecific mitochondrial variation (Hedin 2001). As also found for Appalachian samples, both SNAPP and TR2 show a trend of increasing support for models with increasing numbers of species (Tables 1, 3). GMYC analysis of CO1 data similarly delimits all unique geographic locations as distinct species (Fig. 5 inset). We contend that not all local populations can represent unique species, and instead view this as another example of algorithmic over-splitting.

Based on bootstrap values, nuclear data strongly support the hypothesis that *H.bernardino* is phylogenetically nested within *H.petrunkevitchi* (bootstrap = 100 for all matrices across all analyses; Figs 2, 3; https://doi.org/10.5061/dryad.g1jwstqsd). Acknowledging that bootstrap values can provide an inflated view of support (Lanfear 2018; Minh et al. 2018), we considered several other concordance factor values for this node (gCF = 34.8, sCF = 48.3, Fig. 3). From a gene (individual UCE locus) perspective, of 155 alignments that could have included the branch of interest (gN = 155), 34.84% or ~ 50 alignments (= gCF) showed the Fig. 3 topology, with very few alignments supporting an alternative topology at high frequency (gDF1 = 3.23, gDF2 = 8.39). Similarly, from a site perspective, of ~ 200 decisive sites for the quartet of interest (sN = 199.42), approximately half support the Fig. 3 topology (sCF = 48.28), with fewer supporting alternative resolutions (sDF1 = 18.33, sDF2 = 29.4). Overall, we view these values (see Lanfear 2018), in concert with bootstrap values, as strongly supporting the paraphyly of *H.petrunkevitchi* with respect to *H.bernardino*.

Based on this phylogenomic pattern we see two obvious taxonomic alternatives. The first is to sink *H.bernardino* into a broadly distributed, highly genetically-structured *H.petrunkevitchi*. The second is to elevate and formally describe the distinct genetic lineage that is sister to *H.bernardino*. We prefer and argue for the latter approach, for reasons concisely summarized as follows: 1) *H.bernardino* is already described based on a diagnostic morphology (Catley 1994, and revised diagnosis below), 2) *H.bernardino* is geographically-localized, known only from a single mountain range in southern California, highly disjunct from more northern populations (Fig. 9), and 3) *H.bernardino* is supported both as a distinct nuclear and mitochondrial genetic lineage (Figs 2–5). By these multiple measures of genetic, morphological, and geographic distinctiveness, we view *H.bernardino* as an evolutionary lineage on a unique and independent trajectory. Catley (1994) described *H.bernardino* from the southern section of the San Bernardino mountains of southern California. The few known populations are separated from the southern Sierra Nevada by hundreds of kilometers of mostly inappropriate habitat (lower elevations, fewer granite outcrops), where these spiders (or their distinctive webs) have never been found (Fig. 9). This lack of records includes not only our own extensive field work in the intervening region, but also that of the many arachnologists that have conducted research in California, as well as modern-day tools such as iNaturalist. This sort of geographic disjunction has been found in some other taxa, for example, the iconic *Ensatina* salamander complex, where the geographic disjunction is known as “Bob’s Gaps” (Jackman and Wake 1994). In this instance, separated populations have been described as separate subspecies (*E.eschscholtziiklauberi* in the Tehachapi mountains distinct from *E.eschscholtziicroceater* in the San Bernardino mountains; Jackman and Wake 1994), but *Ensatina* taxonomy is generally regarded as being fairly conservative.

Accepting *H.bernardino* as an independently evolving lineage, the current taxonomy must be updated to reflect unique lineages discovered within *H.petrunkevitchi*. Conservatively, we retain the northern lineages that include the type locality (male holotype of *H.petrunkevitchi* from Cedar Grove, Fresno County = KINGS lineage) as *H.petrunkevitchi*. This lineage is distributed across the Merced, San Joaquin, Kings and Kaweah River basins (Fig. 9). Again, accepting *H.bernardino* as a unique species, our data indicate that populations from the Tule River and Cedar Creek drainage basins need to be recognized as a new species, which we formally describe below. Specimens from these drainage basins represent new locality records and the southern-most known observations of *Hypochilus* spiders in the Sierra Nevada (Fig. 9). More generally, this part of the southern Sierra Nevada is a well-known area of active speciation, with many short-range endemic arthropods and vertebrates (Bond 2012; Jockusch et al. 2012; Papenfuss and Parham 2013; Satler et al. 2013; Leavitt et al. 2015; Emata and Hedin 2016; Starrett et al. 2018; Bennett et al. 2021; Weng et al. 2021). In this regard, discovering a new species in the southern Sierra Nevada is not surprising.

### ﻿Cryptic species concept

We define species as evolutionary lineages on a “unique and independent trajectory”. Following Davis et al. (2021), we consider species to be cryptic “*if they depend on additional sources of data to formulate the delineation hypothesis prior to establishing diagnostic morphological characters*”. This definition applies well to the new species description below, which is motivated by a combination of genetic divergence and uniqueness, phylogenetic pattern (paraphyly w.r.t. *H.bernardino*) and geographic allopatry, which has prompted us to take a closer look at morphology. Additionally, the definition allows for an initial hypothesis of morphological crypsis that does not preclude future downstream studies that in fact find morphological (or other) differences, making the species no longer technically “cryptic”. In many instances, species are morphologically cryptic because of their youth, where morphological divergence has not yet caught up with molecular divergence. But as noted above, the measured mitochondrial differences in *Hypochilus* are among the highest known in spiders (a clade with ~ 50,000 described species), and we hypothesize relatively ancient divergences in the genus. These data and arguments are inconsistent with recent evolutionary divergence, and we instead favor a model of long-term phylogenetic niche conservatism constraining morphological evolution (Fišer et al. 2018), as argued above.

Overall, we view our new cryptic species hypotheses as conservative, consistent with perspectives that genomic data should be interpreted conservatively when describing new species (Coates et al. 2018). In *Hypochilus*, this is particularly true since genomic diagnosability is ubiquitous, and extends to the level of localized populations, as reflected in nuclear and mitochondrial algorithmic delimitations. Our hypotheses below do not recognize all genetically divergent lineages as species and allow for varying degrees of population divergence within described species (e.g., *H.petrunkevitchi*, new species below). In particular, there is evidence that the Yosemite Valley (Merced River) population is on a unique evolutionary trajectory due to its disjunct distribution and because samples from the isolated valley floor routinely fall out as a divergent group in both nuclear and mitochondrial analyses (Figs 2–5). The single sampled population from the San Joaquin drainage is similarly genetically divergent and geographically isolated. This disjunction might be natural, as spiders have never been collected in the apparently suitable granite outcrop habitats between San Joaquin locations and the Yosemite Valley, despite concerted collecting efforts (Fig. 9). In the context of an integrative taxonomic framework, we weigh conservation considerations, extreme geographic allopatry, and well-supported paraphyly as particularly important. Genomic diagnosability is important but not decisive, with morphological diagnosability as least important, again reflecting phylogenetic niche conservatism. Regarding conservation value, we argue that sinking *H.bernardino* into a broadly distributed, highly genetically-structured *H.petrunkevitchi* would immediately decrease the conservation value and importance of the former.

## ﻿Taxonomy

### ﻿ Hypochilus

Taxon classificationAnimaliaAraneaeHypochilidae

Marx, 1888

91C14C56-B238-52D2-B770-EDB0D51FBA5D

#### Diagnosis.

following Forster et al. (1987).

### ﻿ Hypochilus bernardino

Taxon classificationAnimaliaAraneaeHypochilidae

Catley 1994

F0D418C3-6ED9-560D-A505-D286EE0F5ECA

**Figure 10.**
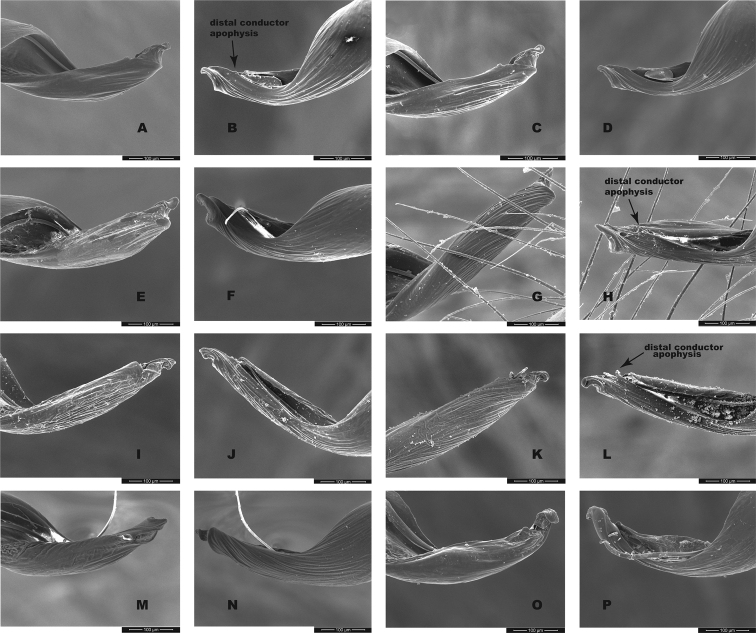
California taxa male palp (conductor) comparison. For each specimen, left panel = prolateral view, right panel = retrolateral view. *H.bernardino***A, B** Mtn Home (SDSU_G2931) **C, D** Mtn Home (SDSU_G2932); *H.xomote***E, F** Alder Creek (SDSU_G2600) **G, H** Tule River (SDSU_G2289); *H.petrunkevitchi* KINGS lineage **I, J** Mill Flat (SDSU_G2554); *H.petrunkevitchi*KAW lineage **K, L** Mineral King Road (SDSU_TAC000192); *H.petrunkevitchi*YOSE lineage **M, N** Yosemite (SDSU_G2568); *H.kastoni***O, P** Ney Springs (SDSU_TAC000191). Distal conductor apophysis highlighted by arrows. Detailed specimen information provided in Suppl. material 2.

**Figure 11.**
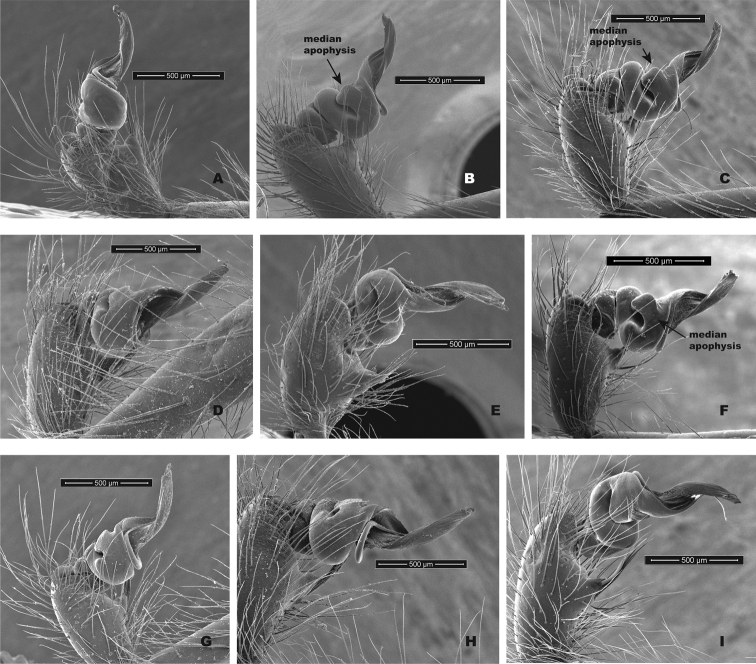
California taxa male palp comparison, prolateral views. *H.kastoni***A** Ney Springs (SDSU_TAC000191); *H.bernardino***B** Mtn Home (SDSU_G2931) **C** Mtn Home (SDSU_G2932); *H.xomote***D** Tule River (SDSU_G2289) **E** Alder Creek (SDSU_G2600) **F** Belknap Springs (SDSU_G2300); *H.petrunkevitchi* KINGS lineage **G** Mill Flat (SDSU_G2554); *H.petrunkevitchi*KAW lineage **H** Mineral King Road (SDSU_TAC000192); *H.petrunkevitchi*YOSE lineage **I** Yosemite (SDSU_G2568). Median apophysis highlighted by arrows. For specimens E and I the bulb has rotated during specimen prep; these two images are retrolateral views, subsequently flipped in Photoshop. Detailed specimen information provided in Suppl. material 2.

**Figure 12.**
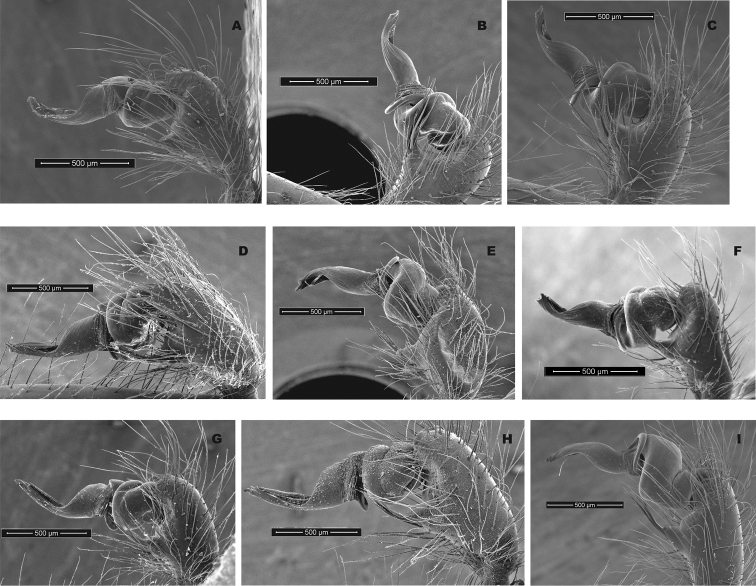
California taxa male palp comparison, retrolateral views. *H.kastoni***A** Ney Springs (SDSU_TAC000191); *H.bernardino***B** Mtn Home (SDSU_G2931) **C** Mtn Home (SDSU_G2932); *H.xomote***D** Tule River (SDSU_G2289) **E** Alder Creek (SDSU_G2600) **F** Belknap Creek (SDSU_G2300); *H.petrunkevitchi* KINGS lineage **G** Mill Flat (SDSU_G2554); *H.petrunkevitchi*KAW lineage **H** Mineral King Road (SDSU_TAC000192); *H.petrunkevitchi*YOSE lineage **I** Yosemite (SDSU_G2568). For specimens E and I the bulb has rotated during specimen prep; these two images are prolateral views, subsequently flipped in Photoshop. Detailed specimen information provided in Suppl. material 2.

**Figure 13.**
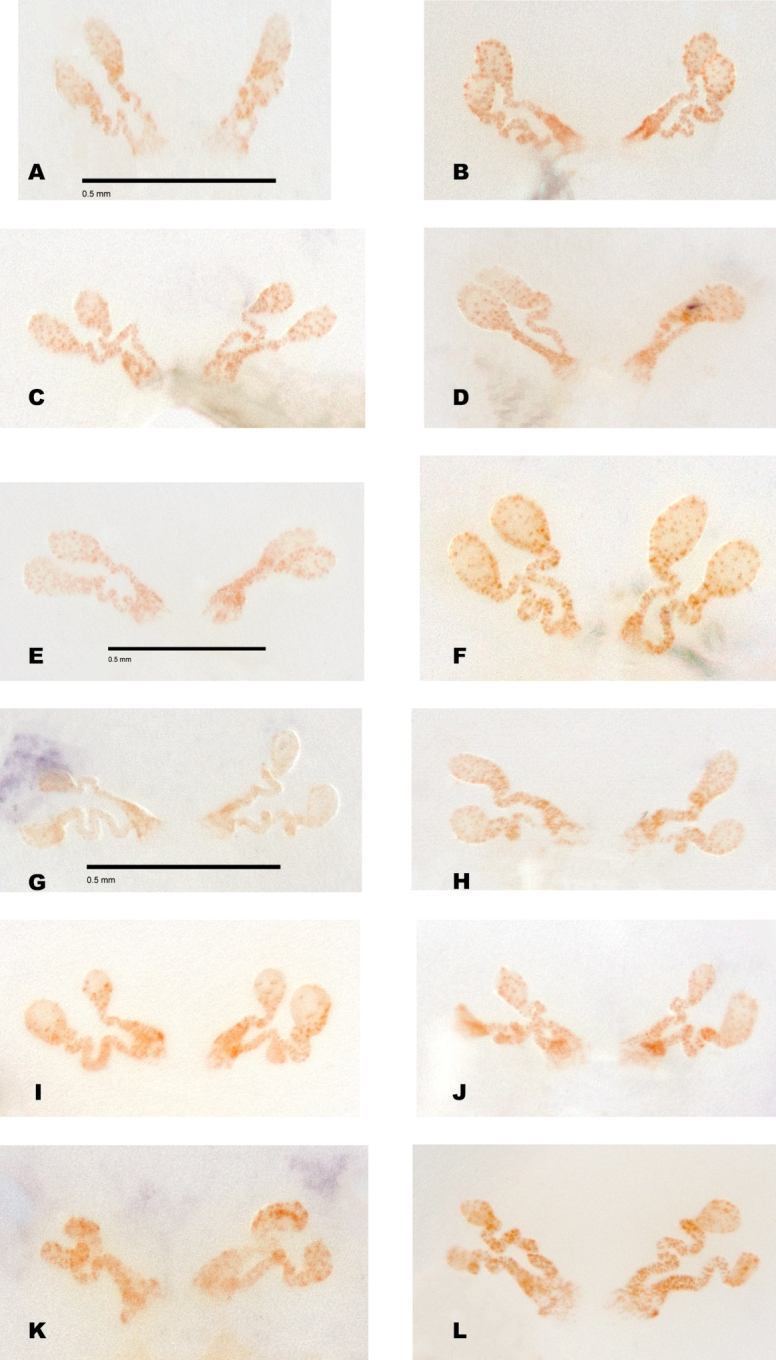
Comparative female spermathecal morphology for California taxa. *H.bernardino***A** Foresee Creek (SDSU_G2893); *H.petrunkevitchi*YOSE lineage **B** Yosemite (SDSU_G2564); *H.xomote***C** Jenny Creek (SDSU_G2477) **D** Windy Creek (SDSU_G2465) **E** Belknap Creek (SDSU_G2296) **F** Alder Creek (SDSU_G2601); *H.petrunkevitchi*KAW lineage **G** Ladybug Trail (SDSU_G2275) **H** Mineral King Road (SDSU_G2485); *H.petrunkevitchi* KINGS lineage **I** Mill Creek (SDSU_G2543) **J** Providence Creek (SDSU_G2508); *H.petrunkevitchi*SAN lineage **K** Snowslide Creek (SDSU_G2557) **L** Balsam Creek (SDSU_G2415). Scale bars shown for select specimens. Detailed specimen information provided in Suppl. material 2.

Figs 9 , 10 , 11 , 12 , 13

Hypochilus petrunkevitchi Gertsch 1958: Forster et al. 1987: 22 (San Bernardino county records).
Hypochilus bernardino Catley 1994: 10, figs 7, 11, 25, 33, 36–39.

#### Material examined.

F from Forsee Creek (SDSU_G2893), Ms from East Fork Mountain Home Creek (SDSU_G2929–2932), see Suppl. material 2.

#### Diagnosis.

Following from the original diagnosis of Catley (1994), we paid closest attention to the length of the PTaL (should be shorter in *H.bernardino*), and the PTW/PTL (should be shorter and more thickened proximally in *H.bernardino*). We found that PTaL overlaps with northern populations (Table 5), and is therefore not diagnostic. The PTW/PTL ratio is generally smaller in *H.bernardino*, but there is some overlap with northern populations, again calling into question the diagnostic value of this character. We did find that the male CdL is consistently shorter in *H.bernardino* (Table 5), and hypothesize this as a new morphological character diagnostic for the species. Again, consistent with a hypothesis of phylogenetic niche conservatism imparting morphological stasis, the species is only weakly morphologically diagnoseable. The disjunct geographic distribution (Fig. 9) and hundreds of diagnostic nucleotide changes (alignments at https://doi.org/10.5061/dryad.g1jwstqsd) can also be used to recognize this species.

**Table 5.** Morphological measurements. PTW/PTL (maximum width of male pedipalpal tibia in retrolateral view/length of tibia in retrolateral view), CdL (male palpal conductor length in retrolateral view), AME (diameter of anterior median eye pupil), PTaL (length of male palpal tarsus in retrolateral view), CTpr (number of promarginal cheliceral teeth), CTre (number of retromarginal cheliceral teeth). Raw measurements provided in Suppl. material 2.

	PTW/PTL	CdL	AME	PTaL	CTpr	CTre
*H.bernardino*	0.253–0.267	0.475–0.50	0.1–0.125	0.82–1.0	4–5	2–3
*H.petrunkevitchi*YOSE lineage	0.259–0.292	0.625	0.10–0.125	0.925–1.0	5	2
*H.petrunkevitchi*KING lineage	0.278	0.60	0.10	0.875	5	2
*H.petrunkevitchi*KAW lineage	0.274–0.307	0.575–0.675	0.10–0.125	0.925–1.15	4–5	2
*H.xomote* sp. nov.	0.256–0.338	0.525–0.575	0.10–0.125	0.775–1.075	4–5	1–2

#### Genetic data.

SRA Accession numbers: SAMN21239435–SAMN21239437.

#### New records.

California, San Bernardino County, San Bernardino Mountains, Camp Creek east of Forest Falls, 34.0760, -116.8876, coll. M. Hedin, 10 July 1993 (SDSU_H0025–0027, 3I). San Bernardino County, San Bernardino Mountains, Hwy 38, tributary into East Fork Mountain Home Creek, in culvert and tunnel under highway, 34.1198, -116.9768, coll. E. Ciaccio, 4 August 2018 (SDSU_G2897–2899, 3I). San Bernardino County, San Bernardino Mountains, Hwy 38, tributary into East Fork Mountain Home Creek, in culvert and tunnel under highway, 34.1198, -116.9768, coll. E. Ciaccio, 27 Sept 2018 (SDSU_G2929–2932, 4M). San Bernardino County, San Bernardino Mountains, Hwy 38, Forsee Creek, along stream and tunnel under highway, 34.1574, -116.9315, coll. E. Ciaccio, 4 August 2018 (SDSU_G2893–2896, F, 3I). See also Suppl. material 2 for locality (including elevation) and natural history information for specimens examined.

#### Remarks.

Catley (1994) hypothesized the following diagnostic features, based on comparisons to near-type locality *H.petrunkevitchi*:

“*The species most closely resembles its sister species Hypochiluspetrunkevitchi in general coloration, eye dimensions, and male pedipalpal morphology. Males can be recognized by the apex of the conductor which is more loosely whorled (fig. 24) than in H.petrunkevitchi, the shorter pedipalpal tarsus, a greatly reduced distal palpal (conductor) apophysis (fig. 25), and a median palpal apophysis that is significantly smaller than H.petrunkevitchi, with no notch (fig. 7). The short palpal tibia is strongly incrassate proximally. Females … are particularly difficult to separate from H.petrunkevitchi females, the former possessing similar but smaller convoluted spermathecal ducts (fig. 11)*.”

Our comparisons of near-type *H.bernardino* to larger samples (Suppl. material 2) of more northern populations in California (not including the distinctive *H.kastoni*) suggests the following character trends. Regarding the shape of the apex of the conductor, we find no consistent difference in tightness of the whorls (Fig. 10), a feature that we also found difficult to characterize. The small distal conductor apophysis is likewise inconsistently absent or barely present across populations (Fig. 10). We also could not discern a consistent difference in the shape of the median palpal apophysis (Figs 11, 12), with our SEM imaged *H.bernardino* specimens appearing much like the original drawings of *H.petrunkevitchi* (Catley 1994: fig. 8) . Differences in the degree of sclerotization at the base of this apophysis also makes the narrowness somewhat subjective to score, at least in some specimens. Finally, we found female spermathecal morphology to be highly conserved (Fig. 13); it is possible that the median ducts are more convoluted in *H.bernardino* than in northern populations, but this difference is subtle given our sampling.

#### Distribution and habitat.

Known only from two primary forks of a single drainage basin (headwaters of Santa Ana River, and Mill Creek, a large tributary of the Santa Ana), south side of the San Bernardino Mountains of southern California (Fig. 9). The Forsee Creek population, near the headwaters of the Santa Ana River, represents a new record for this species. We suspect that additional populations likely exist in the narrow canyons that lead into the Santa Ana River, for example, Bear Creek, Warm Springs Canyon, etc. In our recent collections we have found spiders in webs on large, sheltered granite boulders near streams, and in stream culverts beneath a primary highway.

#### Conservation.

We view *H.bernardino* as a short-range endemic taxon with a precarious future, deserving of special conservation attention and monitoring efforts. Over 25 years ago, Catley (1994) discussed how populations may have been negatively affected by drought. More recently, large fires have burned the forests of the Mountain Home Creek drainage (e.g., 2018 Valley fire). The loss of forest canopy cover is expected to result in fundamental changes in microhabitat conditions, and in concert with increasing global temperatures, calls for continued close monitoring of *H.bernardino* populations.

### ﻿ Hypochilus petrunkevitchi

Taxon classificationAnimaliaAraneaeHypochilidae

Gertsch, 1958

B34D0D3A-7099-5456-9477-C9B209BD694B

Figs 9 , 10 , 11 , 12 , 13

Hypochilus petrunkevitchi Gertsch 1958: 11, figs. 5, 7, 15, 17, 21; Lehtinen 1967: 431, fig. 14; Forster et al. 1987: 21, figs 68–72; Catley 1994: 7, figs. 8, 13, 24.

#### Material examined.

Fs from Ladybug Trail (SDSU_G2275), Mineral King Road (SDSU_G2485), Providence Creek (SDSU_G2508), Mill Creek (SDSU_G2543), Huntington Lake Road (SDSU_G2514, SDSU_G2557), Yosemite Falls (SDSU_G2564); Ms from Atwell-Hockett Trail (SDSU_G2260), Big Fern Springs (SDSU_G2262), Ladybug Trail (SDSU_G2274), Mehrten Creek (SDSU_G2285), Mineral King Road (SDSU_TAC000192), South Fork Kaweah River (SDSU_G2279), Mill Flat (SDSU_G2254), and Yosemite Falls (SDSU_G2568, SDSU_G2569); see Suppl. material 2.

#### Diagnosis.

We found that the male palpal conductor (CdL) is consistently longer in *H.petrunkevitchi* than in both *H.bernardino* and the new species below, although barely for the latter (Table 5), and larger sample sizes might negate this difference. It is clear that all southern Sierran populations retain a very similar morphology, with minor morphological divergence associated with evolution in the southern Transverse ranges (*H.bernardino*).

#### Genetic data.

SRA Accession numbers: SAMN21239424–SAMN21239431.

#### New records.

Merced River drainage (YOSE): California, Mariposa County, Yosemite NP, Big Oak Flat Rd., bridge over Tamarack Creek, 37.7278, -119.7143, coll. E. Ciaccio, M. Hedin, A. Rivera, 29 Sept 2017 (SDSU_G2561–2563, 3I). Mariposa County, Yosemite NP, vic Bridalveil Falls, 37.7167, -119.6519, coll. E. Ciaccio, 3 August 2017 (SDSU_G2515–2518, 4I). Mariposa County, Yosemite NP, near Yosemite Falls, 37.7491, -119.5965, coll. E. Ciaccio, M. Hedin, A. Rivera, 29 Sept 2017 (SDSU_G2564–2566, 2I, 2F, 2M). Mariposa County, Yosemite NP, near Yosemite Falls, 37.7491, -119.5965, coll. M. Hedin, K. Crandall, 27 June 1992 (SDSU_H0015–H0016, 2I). San Joaquin River drainage (SAN): Fresno County, Sierra NF, Huntington Lake Rd., Balsam Creek turnout, 37.1884, -119.2591, coll. E. Ciaccio, 31 July 2017 (SDSU_G2510–2514, 1I, 4F). Fresno County, Sierra NF, Snowslide Creek on Huntington Lake road, 37.2029, -119.2367, coll. E. Ciaccio, 10 Sept 2017 (SDSU_G2557–2560, 1I, 3F). Kings River drainage (KING): California, Fresno County, Sierra NF, McKinley Grove Rd., Bear Creek turnout, 37.0411, -119.1202, coll. E. Ciaccio, B. Hernandez, S. Torres, J. Waters, 24 July 2017 (SDSU_G2557–2560, 4I, 1M). Fresno County, Sierra NF, McKinley Grove Big Trees Area, 37.0224, - 119.1066, coll. E. Ciaccio, 10 Sept 2017 (SDSU_G2555, G2556, 1I, 1F). Fresno County, Bretz Mill, Providence Creek, 37.0427, -119.2371, coll. E. Ciaccio, 31 July 2017 (SDSU_G2505–2509, 2M, 3F). Fresno County, Kings Canyon NP, dam at Sheep Creek on Don Cecil Trail, 36.7840, -118.6784, coll. E. Ciaccio, 9 Sept 2017 (SDSU_G2551, 1I). Fresno County, Kings Canyon NP, Road’s end permit station, Bubbs Creek/Zumwalt Meadow trail jct, 36.7918, -118.5871, coll. E. Ciaccio, 9 Sept 2017 (SDSU_G2549–2550, 1I, 1F). Fresno County, Mill Flat OHV staging area, Mill Flat Creek, 36.7452, -119.0047, coll. E. Ciaccio, 30 July 2017 (SDSU_G2502–2504, 3I). Fresno County, Sequoia NF, Mill Flat OHV Staging Area, 36.7471, -119.0046, coll. E. Ciaccio, 9 Sept 2017 (SDSU_G2552–2554, M, 2F). Fresno County, Sequoia NF, Ten Mile Rd., at bridge N of Hume Lake, 36.7838, -118.9006, coll. E. Ciaccio, 8 Sept 2017 (SDSU_G2546–2548, I, 2F). Fresno County, Sequoia NF, Ten Mile Rd., Landslide Creek turnout, 36.7625, -118.8801, coll. E. Ciaccio, 29 July 2017 (SDSU_G2497–2501, 1I, 2M, 2F). Fresno County, Sequoia NF, Hwy 245 at Mill Creek, ~1 mi S of Hwy 180 Jct, 36.7145, -118.9879, coll. E. Ciaccio, 30 July 2017 (SDSU_G2542–2545, 2I, 2F). Kaweah River drainage (KAW): Tulare County, Sequoia NP, Hwy 198, Big Fern Springs, 36.5382, -118.7751, coll. M. Hedin, 12 July 1993 (SDSU_H0020–H0022, 3I). Tulare County, Sequoia NP Hwy 198, Big Fern Springs, 36.5382, -118.7751, coll. E. Ciaccio, 17 August 2016 (SDSU_G2261–G2265, 1M, 3F, 1I). Tulare County, Sequoia NF, Forest Rte 14S11, Boulder Creek turnout, 36.7342, -118.7736, coll. E. Ciaccio, 29 July 2017 (SDSU_G2492–G2496, 1M, 4F). Tulare County, Sequoia NP, Hwy 198 near Lodgepole CG, Marble Fork Kaweah River, 36.6037, -118.7392, coll. E. Ciaccio, 29 July 2017 (SDSU_G2487–G2491, 4M, 1F). Tulare County, Sequoia NP, Mineral King Rd., Squirrel Creek pullout, 36.4428, -118.7694, coll. E. Ciaccio, 28 July 2017 (SDSU_G2482–G2486, 3M, 1F, 1I). Tulare County, Sequoia NP, Atwell-Hockett Trail, bridge on trail, 36.4584, -118.6564, coll. E. Ciaccio, 16 August 2016 (SDSU_G2256–G2260, 3M, 1F, 1I). Tulare County, Sequoia NP, bridge over Marble Fork on road to Crystal Cave, 36.5759, -118.7860, coll. E. Ciaccio, 17 August 2016 (SDSU_G2266–G2270, 4F, 1I). Tulare County, Sequoia NP, Ladybug Trail, upstream from bridge at start of trail, 36.35005, -118.76238, coll. E. Ciaccio, B. Hernandez, S. Torres, 3 Sept 2016 (SDSU_G2273–G2278, 2M, 3F, 1I). Tulare County, Sequoia NP, Middle Fork Trail, 36.5416, -118.7074, coll. E. Ciaccio, 18 August 2016 (SDSU_G2271, 1I). Tulare County, Sequoia NP, Middle Fork Trail, Mehrten Creek, 36.5457, -118.6920, coll. E. Ciaccio, B. Hernandez, S. Torres, 4 Sept 2016 (SDSU_G2284–G2288, 2M, 2F, 1I). Tulare County, Sequoia NP, Middle Fork Trail, near Mehrten Creek, 36.5456, -118.7036, coll. E. Ciaccio, 16 August 2016 (SDSU_G2272, 1I). Tulare County, Sequoia NP, Mineral King Road, turnout on the road, 36.45346, -118.6923, coll. E. Ciaccio, 18 August 2016 (SDSU_G2251–2255, 5F). Tulare County, Sequoia NP, Mineral King Road, crossing of Redwood Creek, W of Atwell Mill CG, 36.4533, -118.7036, coll. M. Hedin, 24 August 2009 (SDSU_TAC000192- TAC000193, 1F, 1M). Tulare County, Sequoia NP, South Fork Kaweah River, jnct of Cedar Creek and South Fork Kaweah River, 36.3551, -118.7335, coll. E. Ciaccio, B. Hernandez, S. Torres, 4 Sept 2016 (SDSU_G2279–G2283, 1M, 3F, 1I). Tulare County, Sequoia NP, below Atwell Mill CG, along Kaweah River, 36.4584, -118.6561, coll. M. Hedin, 23 August 2009 (SDSU_H0842- H0844, 1F, 1M, 1I). See also Suppl. material 2 for locality (including elevation) and natural history information for specimens examined.

#### Remarks.

Catley (1994) provided no formal diagnosis for *H.petrunkevitchi* (and original diagnoses only compared *H.petrunkevitchi* to the easily distinguishable *H.kastoni*), but offered the following differences from *H.bernardino* in his keys to males and females - length of male PTaL relatively long (0.99–1.10 mm); index of shape of male palpal median apophysis (= vertical height of distal edge of apophysis × length of ventral border of apophysis) large (0.02–0.03) and strongly notched at proximal edge; apex of the conductor in tight whorl with large inwardly directed distal apophysis. Spermathecal bulbs large, diameter of largest not less than 0.11 mm; median ducts of greater length than lateral ducts. We have commented above on the shape of the apex of the male conductor (Fig. 10), the shape of the male palpal median apophysis (Figs 11, 12), and a PTaL which overlaps in length (Table 5). Similarly, we view the relative size of spermathecal bulbs, and relative length of median versus lateral ducts as qualitatively similar (Fig. 13).

#### Distribution and habitat.

*H.petrunkevitchi* was previously known from a handful of locations in the west-central Sierra Nevada, and our work has greatly expanded our distributional knowledge for this species (Fig. 9). All previous taxonomic publications involving this species examined only northern specimens (Kaweah River drainage and northwards), which here retain the name *H.petrunkevitchi*.

#### Conservation.

Of all the basins in the southern Sierra Nevada, the Kaweah and Kings populations appear to occupy the most contiguous habitat, as reflected in both nuclear phylogenies and STRUCTURE results (Figs 2–5, 9). The Yosemite and San Joaquin populations, which are geographically isolated and particularly genetically divergent, deserve close monitoring. While all Yosemite populations lie within the boundaries of Yosemite National Park, this does not strictly assure future persistence. Populations in Yosemite Valley occur only in deep breakdown “caves”, and in our experience spiders are not abundant. Both known San Joaquin populations occur in habitats that have recently burned as part of the devastating 2020 Creek Fire, again likely changing the nature of the canopy structure (and thus microclimatic conditions) in this area.

### ﻿ Hypochilus xomote

Taxon classificationAnimaliaAraneaeHypochilidae

Hedin & Ciaccio sp. nov.

900E01A0-49BF-5B41-97D0-007FED0C0055

http://zoobank.org/7AF45D16-59AC-4E3D-B846-3DD14D4B8BBE

**Figure 14.**
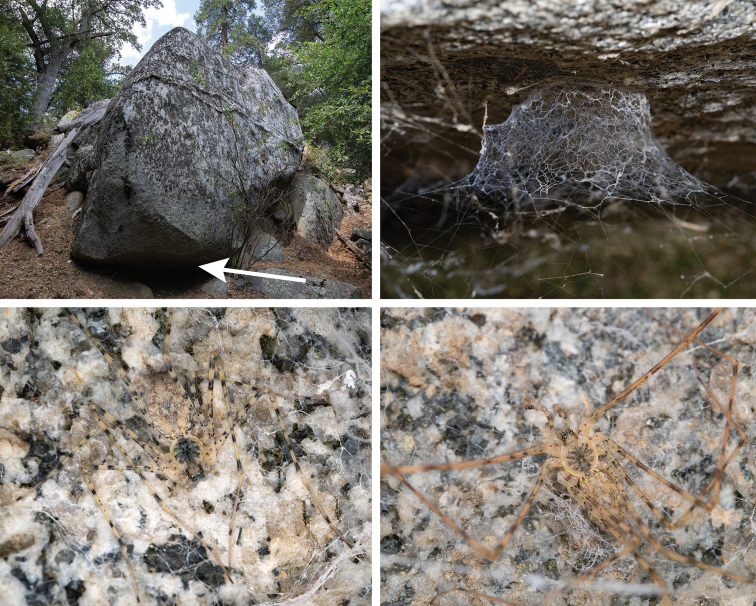
Habitat, web, and live specimen digital images for *H.xomote*. From Kern County, vicinity Alder Creek campground, along Cedar Creek, 5–6 Sept 2020 (see Suppl. material 2) **A** large S-facing granite boulder, on the north side of Cedar Creek. Spider aggregations found in shaded areas, at white arrow **B** web of an adult female **C** image of live adult female **D** image of live adult male.

Figs 9 , 10 , 11 , 12 , 13 , 14

#### Type material.

***Holotype*** male (SDSU_TAC000658) from California, Kern County, upstream of Cedar Creek campground, off Hwy 155, Sequoia National Forest, 35.7508, -118.5807, elevation ~ 1520 meters, coll. M. Hedin, 4 October 2021 (MCH 21_091). Deposited at the University of California Davis Bohart Museum of Entomology. ***Paratype*** females (SDSU_TAC000659, TAC000660) and paratype male (SDSU_TAC000661) from same collecting event (MCH 21_091). Deposited at the Denver Museum of Nature and Science.

#### Etymology.

*xomote*, from the Native American Yowlumni tribal word for south, providing a name for the southern-most known *Hypochilus* populations in the California Sierra Nevada. The X of *xomote* is pronounced as a “breathy, hissy sort-of H” (Vera and Clark 2002). Language translation from the Tule River Yokuts Language Project (Vera and Clark 2002), representing the language of the Yowlumni Yokuts. Members of the larger Yokuts people historically occupied the southern San Joaquin Valley and adjacent Sierran foothills, including the Tule River basin; the Yowlumni occupied a smaller region near the valley outlet of the Kern River (see Fig. 9).

#### Diagnosis.

CdL of intermediate length (Table 5), longer than *H.bernardino* but shorter than *H.petrunkevitchi*, although barely so for geographically adjacent KAW populations of *H.petrunkevitchi*.

#### Genetic data.

SRA Accession numbers: SAMN21239432–SAMN21239434.

#### Description.

**Male holotype** – Total length 7.5. Cephalothorax 3.0 long, 2.4 wide: clypeus 0.10. Eye diameters: AME 0.125, ALE 0.225, PME 0.175, PLE 0.20. Chelicerae pale yellow to white, dusky markings at base; promarginal cheliceral teeth 5, cheliceral formula 52314, retromarginal cheliceral teeth two; one distal, one proximal, both very small. Endites and labium white to pale yellow; sternum with dusky pigmentation, small unpigmented patches circling sparse weak setae; coxae whitish; trochanters with proximal and distal pigmented patches; all legs yellow tan with broken dark annulations on femora and tibiae; pro­lateral proximal aspect of femur 1 with ~ 20 unpigmented weak setae; leg 1 > 20 × length of cephalothorax. Abdomen dorsally pale yellow- white with darker maculations over the entire surface, clothed with sparse hairs, with multiple transverse rows of small weak setae. Palpal tarsus (left) (0.875), palpal tibia short (1.875), thickened proximally (width 0.5), PTW/PTL = 0.267. Conductor length (0.55), conductor tip loosely whorled with very small distal apophysis in retrolateral view. Leg formula 1243; spination (only surfaces bearing spines listed): pedipalpal femur: none; tibia: many dorsal, many prolateral, few to none retrolateral; tarsus: setose with five closely appressed black spines on retrolateral surface of apical spur. Femur I-many prolateral/dorsal; legs II–IV one dorsal proximally. Trichobothrial distribution: all legs with one trichobothria distally on tibia and metatarsus.

**Female paratype** (SDSU_TAC000659): Total length 11.8, cephalothorax 3.8 long, 3.1 wide; clypeus 0.20. Eye diameters: AME 0.175, ALE 0.275, PME 0.225, PLE 0.20. Clypeal area, lateral aspects of head, and foveal area with dusty maculations. Pedipalp pale yellow-white, legs pale yellow-white with femora and tibiae of all legs with broken dark rings and conspicuous dark spots, first leg > 9 × length of cephalothorax. Chelicerae pale yellow, dusky on front proximal surface. Spermathecae with convoluted ducts and relatively large receptacula (e.g., Fig. 13F). Leg formula 1243; spination (only surfaces bearing spines listed): pedipalpal femur: few distal and dorsal; tibia: few dorsal and prolateral, few to none retrolateral; metatarsus: stronger and denser than other pedipalp elements. Femur I-many prolateral/dorsal; legs II–IV one dorsal proximally. Trichobothrial distribution: pedipalpal tibia with a series of dorsal trichobothria; all legs with one trichobothria distally on tibia and metatarsus.

#### Other material examined.

Tule River drainage: California, Tulare County, Sequoia NF, Balch Park Road, Jenny Creek, 36.2843, -118.7335, coll. E. Ciaccio, 28 July 2017 (SDSU G2477–2481, 5F). Tulare County, Sequoia NF, Hwy 190 turnout, Belknap Creek, 36.1534, -118.5977, coll. E. Ciaccio, 23 Sept 2016 (SDSU G2296–G2300, 4F, M). Tulare County, Mountain Home State Forest, Hidden Falls campground, 36.2585, -118.6631, coll. E. Ciaccio, 28 July 2017 (SDSU G2472–G2476, 2F, 3I). Tulare County, Sequoia NF, Hwy 190, McIntyre Creek turnout, 36.1509, -118.5831, coll. E. Ciaccio, 27 July 2017 (SDSU G2467–2471, 3F, 2I). Tulare County, Sequoia NF, North Fork Middle Fork Tule River, 36.2082, -118.6488, coll. E. Ciaccio, 24 Sept 2016 (SDSU G2307–G2311, 4F, M). Tulare County, Sequoia NF, Road 208, North Fork Middle Fork Tule River, 36.1879, -118.6775, coll. E. Ciaccio, 23 Sept 2016 (SDSU G2301–2306, 5F, 1I). Tulare County, Sequoia NF, Hwy 190, Middle Fork Tule River, 36.1556, -118.6688, coll. E. Ciaccio, 23 Sept 2016 (SDSU G2289–2295, 1I, 1M, 5F). Tulare County, Sequoia NF, Forest Route 21S94, Windy Creek turnout, 36.0810, -118.6055, coll. E. Ciaccio, 27 July 2017 (SDSU G2462–2466, 4F, 1I). Cedar Creek drainage: Kern County, Sequoia NF, Alder Creek campground, north side of campground along Cedar Creek, 35.7201, -118.6138, coll. E. Ciaccio & T. Bougie, 26 March 2018 (SDSU G2600–2602, 1M, 2F). Kern County, Sequoia NF, Alder Creek campground, north side of campground along Cedar Creek, 35.7201, -118.6125, coll. M Hedin & O. Hedin, 5 Sept 2020 (SDSU_TAC000657, 2M). Kern County, Hwy 155, Cedar Creek campground, Hwy 155, 35.7500, -118.5810, coll. E. Ciaccio & T. Bougie, 25 March 2018 (SDSU G2596–2599, 4I). Kern County, upstream of Cedar Creek campground, off Hwy 155, Sequoia National Forest, 35.7508, -118.5807, elevation ~ 1520 meters, coll. M. Hedin, 4 October 2021 (MCH 21_091, 1M, 4F).

#### Distribution and habitat.

Known only from the upper Tule River and upper Cedar Creek drainages, at the southern end of the California Sierra Nevada mountains (Fig. 9). We hypothesize that higher elevation xeric ridges (Dennison Mountain ridge in particular) separate the distribution of this species from Kaweah River drainage populations of *H.petrunkevitchi*. Populations of *H.xomote* sp. nov. are predicted to be present in the White River drainage that lies between the Tule and Cedar Creek drainages (Fig. 9), although collecting efforts in this drainage have failed thus far. We have collected these spiders on shaded granite boulders, in mineshafts, and in stream culverts, generally near water along rivers or streams, in conifer or mixed oak/conifer forests (Fig. 14; Suppl. material 2). See also Suppl. material 2 for locality (including elevation) and natural history information for specimens examined.

#### Conservation.

Specimens are more abundant and populations appear more secure in the densely forested and higher elevation / higher latitude Tule River drainage. Specimens are less abundant and populations appear more fragmented in the lower elevation and more southerly Cedar Creek drainage. Recent large fires have occurred in both drainages.

## Supplementary Material

XML Treatment for Hypochilus

XML Treatment for Hypochilus bernardino

XML Treatment for Hypochilus petrunkevitchi

XML Treatment for Hypochilus xomote

## Supplementary materials

Supplementary material 1 Appendix I Data type xslx. file Explanation note Specimens used in phylogenomic analysis. https://binary.pensoft.net/file/647552This dataset is made available under the Open Database License (http://opendatacommons.org/licenses/odbl/1.0/). The Open Database License (ODbL) is a license agreement intended to allow users to freely share, modify, and use this Dataset while maintaining this same freedom for others, provided that the original source and author(s) are credited.Erik Ciaccio, Andrew Debray, Marshal Hedin

Supplementary material 2 Appendix II Data type xslx. file Explanation note Specimens examined and used for study of morphology. https://binary.pensoft.net/file/647553This dataset is made available under the Open Database License (http://opendatacommons.org/licenses/odbl/1.0/). The Open Database License (ODbL) is a license agreement intended to allow users to freely share, modify, and use this Dataset while maintaining this same freedom for others, provided that the original source and author(s) are credited.Erik Ciaccio, Andrew Debray, Marshal Hedin

## References

[B1] Alberti G Coyle FA (1991) Ultrastructure of the primary male genital system, spermatozoa, and spermiogenesis of *Hypochiluspococki* (Araneae, Hypochilidae). Journal of Arachnology 19: 136–149.

[B2] Bennett R Copley C Copley D (2021) *Cybaeus* (Araneae: Cybaeidae): the *aspenicolens* species group of the Californian clade. Zootaxa 4926(2): 224–244. 10.11646/zootaxa.4926.2.4 33756749

[B3] Bernardo J (2011) A critical appraisal of the meaning and diagnosability of cryptic evolutionary diversity, and its implications for conservation in the face of climate change. In: Hodkinson TR Jones MB Waldren S Parnell JAN (Eds) Climate change, ecology and systematics.Cambridge University Press, Cambridge, UK, 380–438. 10.1017/CBO9780511974540.019

[B4] Bickford D Lohman DJ Sodhi NS Ng PK Meier R Bickford D Lohman DJ Sodhi NS Ng PK Meier R Winker K Ingram KK Das I (2007) Cryptic species as a window on diversity and conservation. Trends in Ecology & Evolution 22(3): 148–155. 10.1016/j.tree.2006.11.004 17129636

[B5] Blakey RC Ranney WD (2017) Ancient landscapes of western North America: A geologic history with paleogeographic maps. Springer, Switzerland, 228 pp. 10.1007/978-3-319-59636-5

[B6] Bond JE (2012) Phylogenetic treatment and taxonomic revision of the trapdoor spider genus *Aptostichus* Simon (Araneae, Mygalomorphae, Euctenizidae). ZooKeys 252: 1–209. 10.3897/zookeys.252.3588 PMC3560839 23378811

[B7] Bossert S Danforth BN (2018) On the universality of target‐enrichment baits for phylogenomic research. Methods in Ecology and Evolution 9(6): 1453–1460. 10.1111/2041-210X.12988

[B8] Brewer MS Spruill CL Rao NS Bond JE (2012) Phylogenetics of the millipede genus *Brachycybe* Wood, 1864 (Diplopoda: Platydesmida: Andrognathidae): Patterns of deep evolutionary history and recent speciation. Molecular Phylogenetics and Evolution 64(1): 232–242. 10.1016/j.ympev.2012.04.003 22516430

[B9] Bryant D Bouckaert R Felsenstein J Rosenberg NA RoyChoudhury A (2012) Inferring species trees directly from biallelic genetic markers: bypassing gene trees in a full coalescent analysis. Molecular Biology and Evolution 29(8): 1917–1932. 10.1093/molbev/mss086 22422763 PMC3408069

[B10] Carstens BC Pelletier TA Reid NM Satler JD (2013) How to fail at species delimitation. Molecular Ecology 22(17): 4369–4383. 10.1111/mec.12413 23855767

[B11] Castresana J (2000) Selection of conserved blocks from multiple alignments for their use in phylogenetic analysis. Molecular Biology and Evolution 17: 540–552. 10.1093/oxfordjournals.molbev.a026334 10742046

[B12] Catley KM (1994) Descriptions of new *Hypochilus* species from New Mexico and California with a cladistic analysis of the Hypochilidae (Araneae). American Museum Novitates 3088: 1–27.

[B13] Chambers EA Hillis DM (2019) The multispecies coalescent over-splits species in the case of geographically widespread taxa. Systematic Biology 69(1): 184–193. 10.1093/sysbio/syz042 31180508

[B14] Chifman J Kubatko L (2014) Quartet inference from SNP data under the coalescent model. Bioinformatics 30(23): 3317–3324. 10.1093/bioinformatics/btu530 25104814 PMC4296144

[B15] Coates DJ Byrne M Moritz C (2018) Genetic diversity and conservation units: dealing with the species-population continuum in the age of genomics. Frontiers in Ecology and Evolution 6: 1–13. 10.3389/fevo.2018.00165

[B16] Danecek P Auton A Abecasis G Albers CA Banks E DePristo MA Handsaker RE Lunter G Marth GT Sherry ST McVean G (2011) The variant call format and VCFtools. Bioinformatics 27(15): 2156–2158. 10.1093/bioinformatics/btr330 21653522 PMC3137218

[B17] Davis HR Das I Leaché AD Karin BR Brennan IG Jackman TR Nashriq I Onn Chan K Bauer AM (2021) Genetically diverse yet morphologically conserved: Hidden diversity revealed among Bornean geckos (Gekkonidae: *Cyrtodactylus*). Journal of Zoological Systematics and Evolutionary Research 59: 1113–1135. 10.1111/jzs.12470

[B18] Degnan JH Rosenberg NA (2009) Gene tree discordance, phylogenetic inference and the multispecies coalescent. Trends in Ecology & Evolution 24(6): 332–340. 10.1016/j.tree.2009.01.009 19307040

[B19] Derkarabetian S Starrett J Tsurusaki N Ubick D Castillo S Hedin M (2018) A stable phylogenomic classification of Travunioidea (Arachnida, Opiliones, Laniatores) based on sequence capture of ultraconserved elements. ZooKeys (760): 1–36. 10.3897/zookeys.760.24937 PMC5986891 29872361

[B20] Derkarabetian S Castillo S Koo PK Ovchinnikov S Hedin M (2019) A demonstration of unsupervised machine learning in species delimitation. Molecular Phylogenetics and Evolution 139: 106562. 10.1016/j.ympev.2019.106562 PMC6880864 31323334

[B21] Derkarabetian S Starrett J Hedin M (2022) Using natural history to guide supervised machine learning for cryptic species delimitation with genetic data. Frontiers in Zoology. 10.1186/s12983-022-00453-0 PMC8862334 35193622

[B22] Emata KN Hedin M (2016) From the mountains to the coast and back again: Ancient biogeography in a radiation of short-range endemic harvestmen from California. Molecular Phylogenetics and Evolution 98: 233–243. 10.1016/j.ympev.2016.02.002 26876666

[B23] Evanno G Regnaut S Goudet J (2005) Detecting the number of clusters of individuals using the software STRUCTURE: a simulation study. Molecular Ecology 14(8): 2611–2620. 10.1111/j.1365-294X.2005.02553.x 15969739

[B24] Faircloth BC (2013) illumiprocessor: a trimmomatic wrapper for parallel adapter and quality trimming. 10.6079/J9ILL

[B25] Faircloth BC (2016) PHYLUCE is a software package for the analysis of conserved genomic loci. Bioinformatics 32: 786–788. 10.1093/bioinformatics/btv646 26530724

[B26] Faircloth BC (2017) Identifying conserved genomic elements and designing universal bait sets to enrich them. Methods in Ecology and Evolution 8(9): 1103–1112. 10.1111/2041-210X.12754

[B27] Fernández R Kallal RJ Dimitrov D Ballesteros JA Arnedo MA Giribet G Hormiga G (2018) Phylogenomics, diversification dynamics, and comparative transcriptomics across the spider tree of life. Current Biology 28(9): 1489–1497. 10.1016/j.cub.2018.03.064 29706520

[B28] Fišer C Robinson CT Malard F (2018) Cryptic species as a window into the paradigm shift of the species concept. Molecular Ecology 27(3): 613–635. 10.1111/mec.14486 29334414

[B29] Forster RR Platnick NI Gray MR (1987) A review of the spider superfamilies Hypochiloidea and Austrochiloidea (Araneae, Araneomorphae). Bulletin of the American Museum Natural History 185(1): 1–116. https://digitallibrary.amnh.org/handle/2246/969?show=full

[B30] Fujisawa T Barraclough TG (2013) Delimiting species using single-locus data and the Generalized Mixed Yule Coalescent approach: a revised method and evaluation on simulated data sets. Systematic Biology 62(5): 707–724. 10.1093/sysbio/syt033 23681854 PMC3739884

[B31] Fujisawa T Aswad A Barraclough TG (2016) A rapid and scalable method for multilocus species delimitation using Bayesian model comparison and rooted triplets. Systematic Biology 65(5): 759–771. 10.1093/sysbio/syw028 27055648 PMC4997007

[B32] Garrison NL Rodriguez J Agnarsson I Coddington JA Griswold CE Hamilton CA Hedin M Kocot KM Ledford JM Bond JE (2016) Spider phylogenomics: Untangling the Spider Tree of Life. PeerJ 4: e1719. 10.7717/peerj.1719 PMC4768681 26925338

[B33] Gertsch WJ (1958) The spider family Hypochilidae. American Museum Novitates 1912: 1–28.

[B34] Gertsch WJ (1964) A review of the genus *Hypochilus* and a description of a new species from Colorado (Araneae, Hypochilidae). American Museum Novitates 2203: 1–14.

[B35] Gittenberger E (1991) What about non-adaptive radiation? Biological Journal of the Linnean Society 43(4): 263–272. 10.1111/j.1095-8312.1991.tb00598.x

[B36] Grabherr MG Haas BJ Yassour M Levin JZ Thompson DA Amit I Adiconis X Fan L Raychowdhury R Zeng Q Chen Z (2011) Full-length transcriptome assembly from RNA-Seq data without a reference genome. Nature Biotechnology 29(7): 644–652. 10.1038/nbt.1883 PMC3571712 21572440

[B37] Harvey MS (2002) Short-range endemism among the Australian fauna: some examples from non-marine environments. Invertebrate Systematics 16: 555–570. 10.1071/IS02009

[B38] Harvey MS Rix MG Framenau VW Hamilton ZR Johnson MS Teale RJ Humphreys G Humphreys WF (2011) Protecting the innocent: studying short-range endemic taxa enhances conservation outcomes. Invertebrate Systematics 25(1): 1–10. 10.1071/IS11011

[B39] Harvey MG Smith BT Glenn TC Faircloth BC Brumfield RT (2016) Sequence capture versus restriction site associated DNA sequencing for shallow systematics. Systematic Biology 65: 910–924. 10.1093/sysbio/syw036 27288477

[B40] Hedin MC (2001) Molecular insights into species phylogeny, biogeography, and morphological stasis in the ancient spider genus *Hypochilus* (Araneae: Hypochilidae). Molecular Phylogenetics and Evolution 18: 238–251. 10.1006/mpev.2000.0882 11161759

[B41] Hedin M (2015) High‐stakes species delimitation in eyeless cave spiders (*Cicurina*, Dictynidae, Araneae) from central Texas. Molecular Ecology 24(2): 346–361. 10.1111/mec.13036 25492722

[B42] Hedin M Wood DA (2002) Genealogical exclusivity in geographically proximate populations of *Hypochilusthorelli* Marx (Araneae, Hypochilidae) on the Cumberland Plateau of North America. Molecular Ecology 11: 1975–1988. 10.1046/j.1365-294X.2002.01561.x 12296942

[B43] Hedin M Carlson D Coyle F (2015) Sky island diversification meets the multispecies coalescent–divergence in the spruce‐fir moss spider (*Microhexuramontivaga*, Araneae, Mygalomorphae) on the highest peaks of southern Appalachia. Molecular Ecology 24(13): 3467–3484. 10.1111/mec.13248 26011071

[B44] Hedin M Derkarabetian S Alfaro A Ramírez MJ Bond JE (2019) Phylogenomic analysis and revised classification of atypoid mygalomorph spiders (Araneae, Mygalomorphae), with notes on arachnid ultraconserved element loci. PeerJ 7: e6864. 10.7717/peerj.6864 PMC6501763 31110925

[B45] Heled J Drummond AJ (2010) Bayesian inference of species trees from multilocus data. Molecular Biology and Evolution 27(3): 570–580. 10.1093/molbev/msp274 19906793 PMC2822290

[B46] Herbert PD Ratnasingham S de Waard JR (2003) Barcoding animal life: cytochrome c oxidase subunit 1 divergences among closely related species. Proceedings of the Royal Society of London Series B, Biological Sciences 270(suppl. 1): S96–S99. 10.1098/rsbl.2003.0025 PMC1698023 12952648

[B47] Hoffman RL (1963) A second species of the spider genus *Hypochilus* from eastern North America. American Museum Novitates 2148: 1–8.

[B48] Huff RP Coyle FA (1992) Systematics of *Hypochilussheari* and *Hypochiluscoylei*, two southern Appalachian lampshade spiders (Araneae, Hypochilidae). Journal of Arachnology 20(1): 40–46.

[B49] Hundsdoerfer AK Lee KM Kitching IJ Mutanen M (2019) Genome-wide SNP data reveal an overestimation of species diversity in a group of hawkmoths. Genome Biology and Evolution 11(8): 2136–2150. 10.1093/gbe/evz113 31143925 PMC6685492

[B50] Jackman TR Wake DB (1994) Evolutionary and historical analysis of protein variation in the blotched forms of salamanders of the *Ensatina* complex (Amphibia: Plethodontidae). Evolution 48(3): 876–897. 10.1111/j.1558-5646.1994.tb01369.x 28568256

[B51] Janes JK Miller JM Dupuis JR Malenfant RM Gorrell JC Cullingham CI Andrew RL (2017) The K = 2 conundrum. Molecular Ecology 26(14): 3594–3602. 10.1111/mec.14187 28544181

[B52] Jockusch EL Martínez-Solano I Hansen RW Wake DB (2012) Morphological and molecular diversification of slender salamanders (Caudata: Plethodontidae: *Batrachoseps*) in the southern Sierra Nevada of California with descriptions of two new species. Zootaxa 3190: 1–30. 10.11646/zootaxa.3190.1.1

[B53] Kalyaanamoorthy S Minh BQ Wong TK Von Haeseler A Jermiin LS (2017) ModelFinder: fast model selection for accurate phylogenetic estimates. Nature Methods 14(6): 587–589. 10.1038/nmeth.4285 28481363 PMC5453245

[B54] Kass RE Raftery AE (1995) Bayes factors. Journal of the American Statistical Association 90(430): 773–795. 10.1080/01621459.1995.10476572

[B55] Katoh K Standley DM (2013) MAFFT multiple sequence alignment software version 7: improvements in performance and usability. Molecular Biology and Evolution 30(4): 772–780. 10.1093/molbev/mst010 23329690 PMC3603318

[B56] Keith R Hedin M (2012) Extreme mitochondrial population subdivision in southern Appalachian paleoendemic spiders (Araneae: Hypochilidae: *Hypochilus*), with implications for species delimitation. Journal of Arachnology 40: 167–181. 10.1636/A11-49.1

[B57] Kimura M (1980) A simple method for estimating evolutionary rates of base substitutions through comparative studies of nucleotide sequences. Journal of Molecular Evolution 16(2): 111–120. 10.1007/BF01731581 7463489

[B58] Kopelman NM Mayzel J Jakobsson M Rosenberg NA Mayrose I (2015) Clumpak: a program for identifying clustering modes and packaging population structure inferences across K. Molecular Ecology Resources 15(5): 1179–1191. 10.1111/1755-0998.12387 25684545 PMC4534335

[B59] Kozak KH Weisrock DW Larson A (2006) Rapid lineage accumulation in a non-adaptive radiation: phylogenetic analysis of diversification rates in eastern North American woodland salamanders (Plethodontidae: Plethodon). Proceedings of the Royal Society B: Biological Sciences 273(1586): 539–546. 10.1098/rspb.2005.3326 PMC1560065 16537124

[B60] Lanfear R (2018) Calculating and interpreting gene- and site-concordance factors in phylogenomics. http://www.robertlanfear.com/blog/files/concordance_factors.html

[B61] Lanfear R Calcott B Ho SY Guindon S (2012) PartitionFinder: combined selection of partitioning schemes and substitution models for phylogenetic analyses. Molecular Biology and Evolution 29(6): 1695–1701. 10.1093/molbev/mss020 22319168

[B62] Leaché AD Fujita MK Minin VN Bouckaert RR (2014) Species delimitation using genome-wide SNP data. Systematic Biology 63(4): 534–542. 10.1093/sysbio/syu018 24627183 PMC4072903

[B63] Leavitt DH Bezy RL Crandall KA Sites Jr JW (2007) Multi-locus DNA sequence data reveal a history of deep cryptic vicariance and habitat-driven convergence in the desert night lizard *Xantusiavigilis* species complex (Squamata: Xantusiidae). Molecular Ecology 16: 4455–4481. 10.1111/j.1365-294X.2007.03496.x 17868311

[B64] Leavitt DH Starrett J Westphal MF Hedin M (2015) Multilocus sequence data reveal dozens of putative cryptic species in a radiation of endemic Californian mygalomorph spiders (Araneae, Mygalomorphae, Nemesiidae). Molecular Phylogenetics and Evolution 91: 56–67. 10.1016/j.ympev.2015.05.016 26025426

[B65] Ledford J Derkarabetian S Ribera C Starrett J Bond JE Griswold C Hedin M (2021) Phylo­genomics and biogeography of leptonetid spiders (Araneae: Leptonetidae). Invertebrate Systematics 35(3): 332–349. 10.1071/IS20065

[B66] Li J-N Yan X-Y Lin Y-J Li S-Q Chen H-F (2021) Challenging Wallacean and Linnean shortfalls: *Ectatosticta* spiders (Araneae, Hypochilidae) from China. Zoological Research 42(6): 791–794. 10.24272/j.issn.2095-8137.2021.212 PMC8645878 34704425

[B67] Magalhaes ILF Neves DM Santos FR Vidigal THDA Brescovit AD Santos AJ (2019) Phylo­geny of Neotropical *Sicarius* sand spiders suggests frequent transitions from deserts to dry forests despite antique, broad-scale niche conservatism. Molecular Phylogenetics and Evolution 140: 106569. 10.1016/j.ympev.2019.106569 31362083

[B68] Magalhaes IL Azevedo GH Michalik P Ramírez MJ (2020) The fossil record of spiders revisited: implications for calibrating trees and evidence for a major faunal turnover since the Mesozoic. Biological Reviews 95(1): 184–217. 10.1111/brv.12559 31713947

[B69] Magalhaes IL Porta AO Wunderlich J Proud DN Ramírez MJ Pérez-González A (2021) Taxonomic revision of fossil Psilodercidae and Ochyroceratidae spiders (Araneae: Synspermiata), with a new species of *Priscaleclercera* from mid-Cretaceous Burmese amber, northern Myanmar. Cretaceous Research 121: 104751. 10.1016/j.cretres.2020.104751

[B70] Marshall TL Chambers EA Matz MV Hillis DM (2021) How mitonuclear discordance and geographic variation have confounded species boundaries in a widely studied snake. Molecular Phylogenetics and Evolution 162: 107194. 10.1016/j.ympev.2021.107194 33940060

[B71] Marx G (1888) On a new and interesting spider. Entomologica Americana 4: 160–162.

[B72] Mason NA Fletcher NK Gill BA Funk WC Zamudio KR (2020) Coalescent-based species delimitation is sensitive to geographic sampling and isolation by distance. Systematics and Biodiversity 18(3): 269–280. 10.1080/14772000.2020.1730475

[B73] Minh B Hahn M Lanfear R (2018) New methods to calculate concordance factors for phylogenomic datasets. Molecular Biology and Evolution 37(9): 2727–2733. 10.1093/molbev/msaa106 PMC7475031 32365179

[B74] Niemiller ML Near TJ Fitzpatrick BM (2012) Delimiting species using multilocus data: diagnosing cryptic diversity in the southern cavefish, *Typhlichthyssubterraneus* (Teleostei: Amblyopsidae). Evolution: International Journal of Organic Evolution 66(3): 846–866. 10.1111/j.1558-5646.2011.01480.x 22380444

[B75] Nguyen LT Schmidt HA Von Haeseler A Minh BQ (2015) IQ-TREE: A fast and effective stochastic algorithm for estimating maximum-likelihood phylogenies. Molecular Biology and Evolution 32(1): 268–274. 10.1093/molbev/msu300 25371430 PMC4271533

[B76] Paradis E Schliep K (2019) ape 5.0: an environment for modern phylogenetics and evolutionary analyses in R. Bioinformatics 35: 526–528. 10.1093/bioinformatics/bty633 30016406

[B77] Papenfuss TJ Parham JF (2013) Four new species of California legless lizards (*Anniella*). Breviora 536(1): 1–17. 10.3099/MCZ10.1

[B78] Pfenninger M Schwenk K (2007) Cryptic animal species are homogeneously distributed among taxa and biogeographical regions. BMC Evolutionary Biology 7(1): e121. 10.1186/1471-2148-7-121 PMC1939701 17640383

[B79] Pritchard JK Stephens M Donnelly P (2000) Inference of population structure using multilocus genotype data. Genetics 155(2): 945–959. 10.1093/genetics/155.2.945 10835412 PMC1461096

[B80] Rambaut A Drummond AJ Xie D Baele G Suchard MA (2018) Posterior summarization in Bayesian phylogenetics using Tracer 1.7. Systematic Biology 67(5): 901–904. 10.1093/sysbio/syy032 29718447 PMC6101584

[B81] Ramírez MJ Magalhaes IL Derkarabetian S Ledford J Griswold CE Wood HM Hedin M (2021) Sequence capture phylogenomics of true spiders reveals convergent evolution of respiratory systems. Systematic Biology 70(1): 14–20. 10.1093/sysbio/syaa043 32497195

[B82] Reilly SB Wake DB (2015) Cryptic diversity and biogeographical patterns within the black salamander (*Aneidesflavipunctatus*) complex. Journal of Biogeography 42(2): 280–291. 10.1111/jbi.12413

[B83] Satler JD Carstens BC Hedin M (2013) Multilocus species delimitation in a complex of morphologically conserved trapdoor spiders (Mygalomorphae, Antrodiaetidae, *Aliatypus*). Systematic Biology 62(6): 805–823. 10.1093/sysbio/syt041 23771888

[B84] Schönhofer AL McCormack M Tsurusaki N Martens J Hedin M (2013) Molecular phylogeny of the harvestmen genus *Sabacon* (Arachnida: Opiliones: Dyspnoi) reveals multiple Eocene–Oligocene intercontinental dispersal events in the Holarctic. Molecular Phylogenetics and Evolution 66(1): 303–315. 10.1016/j.ympev.2012.10.001 23085535

[B85] Singhal S Hoskin CJ Couper P Potter S Moritz C (2018) A framework for resolving cryptic species: a case study from the lizards of the Australian wet tropics. Systematic Biology 67(6): 1061–1075. 10.1093/sysbio/syy026 29635540

[B86] Stamatakis A (2014) RAxML version 8: a tool for phylogenetic analysis and post-analysis of large phylogenies. Bioinformatics 30(9): 1312–1313. 10.1093/bioinformatics/btu033 24451623 PMC3998144

[B87] Starrett J Derkarabetian S Hedin M Bryson RW McCormack JE Faircloth BC (2017) High phylogenetic utility of an ultraconserved element probe set designed for Arachnida. Molecular Ecology Resources 17(4): 812–823. 10.1111/1755-0998.12621 27768256

[B88] Starrett J Hayashi C Derkarabetian D Hedin M (2018) Cryptic elevational zonation in trapdoor spiders (Araneae, Antrodiaetidae, *Aliatypusjanus* complex) from the California southern Sierra Nevada. Molecular Phylogenetics and Evolution 118: 403–413. 10.1016/j.ympev.2017.09.003 28919504

[B89] Stockman AK Bond JE (2007) Delimiting cohesion species: extreme population structuring and the role of ecological interchangeability. Molecular Ecology 16(16): 3374–3392. 10.1111/j.1365-294X.2007.03389.x 17688540

[B90] Sukumaran J Knowles LL (2017) Multispecies coalescent delimits structure, not species. Proceedings of the National Academy of Sciences 114(7): 1607–1612. 10.1073/pnas.1607921114 PMC5320999 28137871

[B91] Swofford DL (2003) PAUP*. Phylogenetic Analysis Using Parsimony (*and Other Methods). Version 4. Sinauer Associates, Sunderland, Mass.

[B92] Talavera G Castresana J (2007) Improvement of phylogenies after removing divergent and ambiguously aligned blocks from protein sequence alignments. Systematic Biology 56: 564–577. 10.1080/10635150701472164 17654362

[B93] Templeton A Shaw K Routman E Davis S (1990) The genetic consequences of habitat fragmentation. Annals of the Missouri Botanical Garden 77(1): 13–27. 10.2307/2399621

[B94] Van der Auwera GA Carneiro MO Hartl C Poplin R Del Angel G Levy‐Moonshine A Jordan T Shakir K Roazen D Thibault J Banks E (2013) From FastQ data to high‐confidence variant calls: the genome analysis toolkit best practices pipeline. Current Protocols in Bioinformatics 43(1): 11–10. 10.1002/0471250953.bi1110s43 25431634 PMC4243306

[B95] Vera A Clark PL (2002) English-Yowlumni dictionary. Tule River Yokuts Language Project. https://tulerivertribe-nsn.gov/language/

[B96] Vieites DR Min MS Wake DB (2007) Rapid diversification and dispersal during periods of global warming by plethodontid salamanders. Proceedings of the National Academy of Sciences 104(50): 19903–19907. 10.1073/pnas.0705056104 PMC2148395 18077422

[B97] Weng YM Kavanaugh DH Schoville SD (2021) Drainage basins serve as multiple glacial refugia for alpine habitats in the Sierra Nevada Mountains, California. Molecular Ecology 30(3): 826–843. 10.1111/mec.15762 33270315

[B98] Wunderlich J (2008) The dominance of ancient spider families of the Araneae: Haplogynae in the Cretaceous, and the late diversification of the advanced ecribellate spiders of the Entelegynae after the Cretaceous-Tertiary boundary extinction events, with descriptions of new families. Beiträge zur Araneologie 5: 524–674, 802–813.

[B99] Zerbino D Birney E (2008) Velvet: algorithms for de novo short read assembly using de Bruijn graphs. Genome Research 18(5): 821–829. 10.1101/gr.074492.107 18349386 PMC2336801

